# Unraveling potent therapeutic potentials of tanshinones, lipophilic abietane diterpenes: a holistic literature review

**DOI:** 10.1007/s00210-026-05664-4

**Published:** 2026-07-20

**Authors:** Shymaa I. A. Abdel-dayem, Rania M. Kamal, Heba R. Ghaiad, Mariam K. Ahmed, Aya H. Eid, Ahmed M. El-Dessouki, Ola Elazazy, Fatma Saeed EL-Tokhy, Sally A. Fahim, Riham A. El-Shiekh, Eman M. Abd Elghany

**Affiliations:** 1 Pharmacognosy Department, Faculty of Pharmacy, Cairo University, Kasr El-Aini Street, Cairo, 11562 Egypt; 2 Department of Biochemistry, Faculty of Pharmacy, Cairo University, Cairo, 11562 Egypt; 3 Pharmacology and Toxicology Department, Faculty of Pharmacy, Heliopolis University, Cairo, Egypt; 4 Pharmacology and Toxicology Department, Faculty of Pharmacy, 6Th of October City, Ahram Canadian University, Giza, 12566 Egypt; 5 Department of Biochemistry, Faculty of Pharmacy, Badr University in Cairo (BUC), Badr City, Cairo, 11829 Egypt; 6 Department of Pharmaceutics and Industrial Pharmacy, Faculty of Pharmacy, Badr University in Cairo (BUC), Badr CityCairo, 11829 Egypt; 7 Department of Biochemistry, School of Pharmacy, Newgiza University (NGU), Km 22 Cairo-Alexandria Desert Road, P.O. Box 12577, NewgizaGiza, Egypt

**Keywords:** Tanshinones, Drug discovery, Biological activities, Phytochemistry

## Abstract

Tanshinones, a class of lipophilic abietane-type diterpenoid, represents the major bioactive constituents of *Salvia miltiorrhiza* Bunge (Danshen), a key herb in traditional Chinese medicine with growing therapeutic importance. This review offers an in-depth overview of tanshinones from phytochemical and pharmacological perspectives. The review outlines the biosynthetic pathway, highlights the key synthetic approaches, and provides a detailed chemical and stereochemical characterization of the major tanshinones isomers. Pharmacologically, tanshinones exhibit broad, multi-system activities including antioxidant and anti-inflammatory effects, cardiovascular and hepatorenal protection, antitumor activity, immunomodulatory effects, and therapeutic benefits across neurological, metabolic, skeletal, respiratory, and infectious diseases. Despite their broad therapeutic potential, progression of tanshinones toward clinical application is restricted by low oral bioavailability, poor aqueous solubility, and rapid metabolism. To overcome these barriers, novel drug-delivery strategies including lipid nanocapsules, liposomes, polymeric nanoparticles, solid liquid nanoparticles, microemulsions, and nanoemulsions significantly improve solubility, stability, and pharmacokinetics. This review integrates the current evidence to guide future optimization and mechanistic exploration and support the development of innovative nanoformulations for next-generation tanshinone-based therapies.

**Supplementary Information:**

The online version contains supplementary material available at https://doi.org/10.1007/s00210-026-05664-4.

## Introduction

Natural compounds have gained significant therapeutic potential by serving as foundational templates for drug discovery (Azzam et al. 2025; Mustafa et al. 2025; El-Dessouki et al. 2026). Tanshinones, a chemical class of lipophilic abietane-type diterpenoid quinones, were first isolated in 1934 from the deep red (rhizomes) roots of *Salvia miltiorrhiza* Bunge (family Lamiaceae), commonly known as Danshen or Tanshen in traditional Chinese medicine (Pan et al. 2002; Huang et al. 2020). Further investigation led to the isolation and identification of more than 40 tanshinone derivatives and structurally related analogues from *S. miltiorrhiza* Bunge (Pan et al. 2001; Zhang et al. 2012; Huang et al. 2020). Key representatives include tanshinone I, tanshinone IIA, isotanshinone I, tanshinone IIB, dihydrotanshinone I, methyltanshinone, cryptotanshinone, and isocryptotanshinone I and II (Pan et al. 2001, 2002). Additionally, numerous tanshinones and structurally related compounds have been isolated from several *Salvia* species distributed across China, Korea, Japan, and Mongolia (Zhang et al. 2012) as well as from the Iranian medicinal herb *Perovskia abrotanoides* (Sairafianpour et al. 2001). Based on their core skeleton, tanshinones can be categorized into two structural subclasses including phenanthro[3,2-*b*]furan-7,11-diones (e.g., tanshinone I) and phenanthro[1,2-*b*]furan-10,11-diones (tanshinone IIA) (Huang et al. 2020). Tanshinones are the principal bioactive components responsible for the biological and pharmacological activities of *S. miltiorrhiza (*Huang et al. 2020*).* Their distinctive structures enable them to interact through multiple significant biological pathways (Sairafianpour et al. 2001; Cai et al. 2016; Huang et al. 2020). As reported, tanshinones are able to downregulate many inflammatory cascades and simultaneously trigger cellular antioxidant defense mechanisms (Huang et al. 2020). Moreover, tanshinones exert regulatory effects on varying oncogenic signaling pathways *via* the inhibition of cancer cell proliferation and growth, the induction of cell cycle arrest and apoptosis, and the suppression of migration, invasion, and metastatic potential of cancer cells (Sairafianpour et al. 2001; Cai et al. 2016; Huang et al. 2020; Fang et al. 2021). Their therapeutic scope also extends to manage various cardio-cerebrovascular diseases (Cai et al. 2016). Additionally, they confer a significant hepatoprotective action versus both disease associated and drug-induced liver injury (Yin et al. 2008; Li et al. 2023), besides mitigating kidney toxicity (Li et al. 2022; Chen et al. 2023). Tanshinones also display broad anti-infective properties including anti-malarial and leishmanicidal activities (Sairafianpour et al. 2001), antiviral effect (Park et al. 2012; Elebeedy et al. 2021), and antibacterial activity (Wang et al. 2015b). Although tanshinones demonstrate extensive pharmacological effects, its clinical application is greatly hindered by poor aqueous solubility and low oral bioavailability (Cai et al. 2016). To overcome this limitation, numerous studies have explored the incorporation of tanshinones into nanoparticles, liposomes, microemulsions, pellets, and micellar formulations to enhance its solubility and therapeutic performance (Cai et al. 2016). In this review, we provide a concise overview of the biosynthetic pathway, outline the key synthetic approaches, and present a detailed chemical and stereochemical characterization of the major tanshinone isomers. Furthermore, we summarize the available biological activities of tanshinones and highlight their therapeutic potential as promising candidates for drug development, with the aim of offering evidence-based conclusion for future pharmacological investigations.

## Scope and boundary delineation of the review

To maintain a rigorous and sharply focused thematic boundary, the scope of this review is strictly confined to the four primary naturally occurring lipophilic abietane diterpenoid quinone monomers isolated from the roots and rhizomes of *Salvia miltiorrhiza* Bunge (Danshen): tanshinone I, tanshinone IIA, cryptotanshinone, and dihydrotanshinone I. Minor synthetic analogues, water-soluble derivatives (such as sodium tanshinone IIA sulfonate), and highly modified hydroquinone structures are included only when directly relevant to expanding structure–activity relationship (SAR) models or highlighting clinical translation.

The rationale for selecting the specific multi-system subtopics discussed herein is grounded in a stringent inclusion protocol: each physiological module must possess documented preclinical evidence demonstrating that the target monomer successfully crosses relevant cellular or tissue barriers to interact with master metabolic, protective, or immunological signaling networks. By focusing exclusively on these high-confidence therapeutic axes, this review provides a systematic evaluation of how these lipophilic scaffolds bridge the gap between traditional phytomedicine and modern molecular pharmacology.

### Search strategy and selection criteria

To ensure methodological transparency and high scholarly currency, a structured literature search was conducted across PubMed/MEDLINE, Web of Science, Embase, Scopus, and the Egyptian Knowledge Bank databases for articles published up to April 2026. The search strategy utilized Boolean operators to pair primary chemical descriptors with specific pathological frameworks using the following string: ("Tanshinone IIA" OR "Cryptotanshinone" OR "Tanshinone I" OR "Dihydrotanshinone I") AND ("liver fibrosis" OR "hepatotoxicity" OR "NASH" OR "osteoporosis" OR "bone remodeling" OR "cathepsin K" OR "Alzheimer's disease" OR "cerebral ischemia" OR "Parkinson's disease" OR "obesity" OR "type 2 diabetes" OR "diabetic nephropathy" OR "psoriasis" OR "keratinocyte" OR "dermatitis").

The retrieved literature was filtered using a stringent inclusion and exclusion protocol to ensure the critical integration of high-quality data. Studies were strictly included if they met the following criteria: (1) original, peer-reviewed full-text experimental articles or clinical trials; (2) investigations focusing explicitly on at least one of the four principal native lipophilic abietane monomers or validated derivatives; and (3) configurations providing clear mechanistic data regarding upstream master signaling networks, cell-cycle regulators, or phenotypic tissue adaptations. Conversely, literature was excluded if it consisted of abstracts without full-text access, conference proceedings, or informal editorials, or if the study focused exclusively on crude, uncharacterized multi-herbal decoctions where individual compounds could not be isolated. Reference management software was used to remove duplicate records, resolve formatting inconsistencies, and ensure rigorous indexing.

## Phytochemistry point of view

### Natural sources

Tanshinones are predominantly natural secondary metabolites produced in the roots and rhizomes of plants in the genus *Salvia*, with *S. miltiorrhiza* Bunge (red sage or Danshen) being the primary and most intensively studied botanical source (Baillie And Thomson 1968). In traditional Chinese medicine, Danshen has been valued for centuries for its medicinal properties, and modern phytochemical investigations have confirmed that its lipophilic diterpenoid constituents are dominated by various tanshinones, including tanshinone I, tanshinone IIA, cryptotanshinone, and dihydrotanshinone I as major compounds, along with numerous minor analogues and derivatives identified through chromatographic and spectroscopic methods (Wang et al. 2020b). These compounds accumulate mainly in the peripheral cortex of Danshen roots, where they contribute to the plant’s characteristic pharmacological profile and have been identified in over 30 distinct diterpenoid tanshinones isolated from *S. miltiorrhiza* and related *Salvia* species. In addition to *S. miltiorrhiza*, related species such as *Salvia castanea*, *Salvia bowleyana*, and other congeners may also biosynthesize tanshinone-type diterpenoids, although with variable profiles and concentrations, highlighting both the taxonomic specificity and ecological influence on natural tanshinone distribution (Zhao et al. 2011).

### Biosynthesis

Biosynthesis of tanshinones including tanshinone I, tanshinone IIA, tanshinone IIB, dihydrotanshinone I, cryptotanshinone, neocryptotanshinone II, 1,2-dihydrotanshinquinone, tanshinone III, isocryptotanshinone, 12-*O*-methylneocryptotanshinone II, and neo-tanshinlactone in *S. miltiorrhiza* proceeds through an intricate, multi-stage terpenoid pathway that integrates both the methylerythritol 4-phosphate pathway in plastids and the mevalonate pathway in the cytosol to supply the universal five-carbon building blocks, isopentenyl diphosphate and dimethylallyl diphosphate. These units are condensed by geranylgeranyl diphosphate synthase to form geranylgeranyl diphosphate, the key diterpenoid precursor, which is then cyclized by copalyl diphosphate synthase and kaurene synthase-like enzymes to yield miltiradiene, the core hydrocarbon scaffold of tanshinones. Subsequent oxidative tailoring is catalyzed by a group of cytochrome P450 monooxygenases (e.g., CYP76AH1, CYP76AH3, CYP76AK1) and other modifying enzymes, including dehydrogenases and dioxygenases that introduce oxygen functionalities and rearrangements, ultimately giving rise to the structurally diverse tanshinone congeners observed in nature. Regulatory layers involving transcription factors such as myeloblastosis (SmMYBs) and signal molecules like methyl jasmonate further modulate the expression of these biosynthetic genes, thereby influencing tanshinone accumulation in plants. Extensive transcriptomic, genetic, and biochemical studies have delineated many of these genes and their roles in the pathway, making *S. miltiorrhiza* a model system for diterpenoid biosynthesis research (Ma et al. 2015a; Zhou et al. 2017; Contreras et al. 2019; Zhu et al. 2024). Collectively, tanshinone biosynthesis can be summarized as a sequential process involving isoprenoid precursor formation, diterpene cyclization, and extensive oxidative tailoring, resulting in the structural diversification of tanshinone I, tanshinone IIA, cryptotanshinone, and related analogues.

### Synthesis

The synthesis of tanshinones has attracted considerable attention due to their diverse pharmacological activities and limited availability from natural sources. Structurally, these abietane-type diterpenoid quinones share a common phenanthrene or phenanthrene-like core with varying degrees of oxidation, unsaturation, and functional group substitution, which poses significant synthetic challenges. Consequently, both total and semi-synthetic strategies have been developed to efficiently construct the polycyclic framework while enabling late-stage diversification. In a more specific way, tanshinone I, tanshinone II, and cryptotanshinone have been synthesized *via* multiple routes (Inouye And Kakisawa 1969). The methodology uses specific starting materials, such as 3-methylbenzofuran-4,7-quinone and 6,6-dimethyl-l-vinylcyclohexene for tanshinone II and cryptotanshinone, quinone X, and *o*-methylstyrene for tanshinone I. Tanshinone IIA has been synthesized through base-mediated furan ring formation and acyloin condensation (Huang et al. 2020). A novel total synthesis of tanshinone I has been reported by the use of the Diels–Alder reaction in three steps (Wu et al. 2017). Tanshinone I was synthesized from a ubiquitous ene intermediate in one to two steps. This critical intermediate was derived from (±)-tanshinol B, which was synthesized in 50% overall yield over 3 steps using an ultrasound-promoted cycloaddition as a key step (Wang et al. 2018). Cryptotanshinone was synthesized as early as 1968–1969 (Baillie And Thomson 1968). The synthesis process involves specific chemical reactions and transformations, such as the conversion of a 2-hydroxy-1,4-quinone into a dihydrofurano-*o*-quinone and subsequent dehydrogenation steps. Overall, advances in tanshinone synthesis have not only improved access to these bioactive molecules but have also facilitated structure–activity relationship studies and the development of novel analogues with enhanced therapeutic potential.

### Chemistry of the compounds and structure elucidation of different isomers

**Fig. 1:**
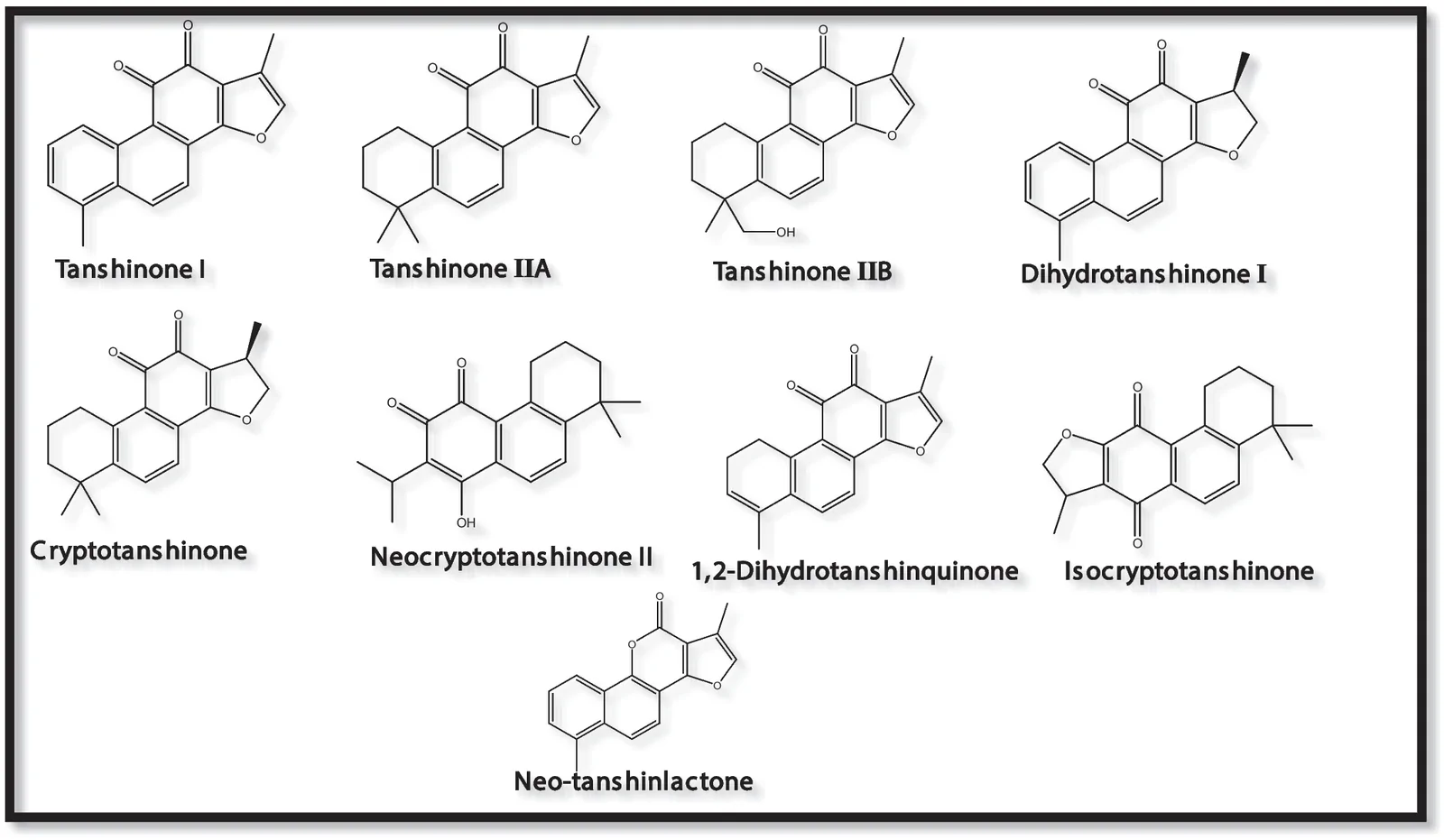
Chemical structures of tanshinone I, tanshinone IIA, tanshinone IIB, dihydrotanshinone I, cryptotanshinone, neocryptotanshinone II, 1,2-dihydrotanshinquinone, isocryptotanshinone, and neo-tanshinlactone

The lipophilic constituents tanshinone I, tanshinone IIA, tanshinone IIB, dihydrotanshinone I, cryptotanshinone, neocryptotanshinone II, 1,2-dihydrotanshinquinone, isocryptotanshinone, and neo-tanshinlactone belong to the abietane-type diterpenoid quinones predominantly isolated from *S. miltiorrhiza* (Danshen). Structurally, these compounds share a phenanthrene-like fused ring system derived from the abietane skeleton, frequently incorporating *ortho*-quinone or furan-quinone moieties, which are responsible for their characteristic red–orange coloration and pronounced redox activity (Fig. 1).

Structurally, variations among these derivatives mainly arise from differences in oxidation state, double-bond saturation, methyl substitution, and lactone formation, giving rise to quinone, hydroquinone, or lactone derivatives such as neo-tanshinlactone. Spectroscopic characterization of these compounds has been extensively established using ^1^H- and ^13^C-NMR, IR spectroscopy, and LC–MS techniques. In general, the ^1^H-NMR spectra of tanshinones display diagnostic aromatic proton resonances within *δ* 6.5–8.0 ppm, together with methyl singlets around δ 1.2–2.3 ppm corresponding to the tertiary methyl groups of the abietane skeleton. Meanwhile, ^13^C-NMR spectra typically exhibits signals for conjugated quinone carbonyl carbons at δ 175–185 ppm, aromatic carbons between *δ* 120 and 160 ppm, and aliphatic carbons in the *δ* 15–40-ppm region. For instance, tanshinone IIA (C₁₉H₁₈O₃) shows characteristic signals at *δ*C 183.8 and 175.9 ppm attributable to quinone carbonyl carbons, along with aromatic signals between *δ*C 120 and 150 ppm, confirming the conjugated diterpenoid quinone system (Dang et al. 2018). Similarly, LC–MS analyses reveal protonated molecular ions such as *m*/*z* 277 [M + H]⁺ for tanshinone I, *m/z* 279 [M + H]⁺ for dihydrotanshinone I, *m/z* 295 [M + H]⁺ for tanshinone IIA, and *m/z* 297 [M + H]⁺ for cryptotanshinone, accompanied by characteristic fragmentation ions produced through cleavage of the phenanthrene-quinone scaffold. Infrared spectra further support the structural assignment, typically displaying strong absorption bands at ~ 1660–1680 cm⁻^1^ corresponding to conjugated quinone C = O stretching, while aromatic C = C vibrations appear near 1580–1620 cm⁻^1^. Additionally, LC–MS/MS and HPLC methods employing reversed-phase C18 columns with acetonitrile–water mobile phases are widely used for simultaneous separation and quantification of major tanshinones including tanshinone I, tanshinone IIA, cryptotanshinone, and dihydrotanshinone I in *S. miltiorrhiza* extracts (Liu et al. 2010). Overall, the combination of NMR, IR, and LC–MS spectral data provides reliable structural confirmation of these abietane diterpenoid quinones and facilitates differentiation among closely related derivatives such as neocryptotanshinone and isocryptotanshinone based on subtle variations in substitution patterns and oxidation states.

### Extraction methods

#### Extraction at room temperature

The isolation of tanshinones (tanshinone I, tanshinone IIA, cryptotanshinone) was conducted using 10 g of *S. miltiorrhiza* Bunge root material, which was macerated in 100 mL of ethanol at 25 °C with intermittent stirring. The extraction yield is time dependent, prolonged room-temperature maceration enhanced the recovery of tanshinones, resulting in an increased yield over a 24-h period (Pan et al. 2002).

#### Heat reflux extraction

Heat reflux extraction of tanshinone I, tanshinone IIA, and cryptotanshinone was performed by macerating 10 g of *S. miltiorrhiza* Bunge roots in 100 mL of ethanol within a 250-mL flask, using a temperature-controlled water bath, with continuous mechanical stirring. The highest extraction yields were observed when using 95% and 100% (v/v) ethanol, at 45 and 60 min, respectively. Prolonged extraction beyond these time points resulted in a decline in yield (Pan et al. 2002). Temperature-dependent analysis revealed a non-linear biphasic response between water bath temperature and extraction efficiency. Tanshinone yields increased steadily as temperature rose up to 65 °C for both ethanol concentrations. However, a pronounced decline in extraction yield occurred at 70 °C, likely resulting from thermal decomposition of these thermolabile diterpenoids. Intriguingly, further elevation of the temperature to 80 °C restored and even maximized extraction yields, as accelerated mass transfer rates apparently compensated for concurrent decomposition losses (Pan et al. 2002).

#### Ultrasonic-assisted extraction (UAE)

To optimize the isolation of tanshinones (tanshinone I, tanshinone IIA, cryptotanshinone) using ultrasonic-assisted extraction, 10 g *S. miltiorrhiza* Bunge root powder was suspended in 100 mL of ethanol and subjected to continuous sonication at temperatures below 70 °C to prevent thermal decomposition. The maximum extraction yield was achieved after 75 min of sonication (Pan et al. 2002; Fikri et al. 2022). The efficiency of UAE of tanshinones was significantly improved by pre-treatment with imidazolium-based ionic liquid surfactants bearing alkyl chains of 10–16 carbons (C₁₀mimBr to C₁₆mimBr) (Wu et al. 2009).

#### Soxhlet extraction

Tanshinone I, tanshinone IIA, and cryptotanshinone were isolated from *S. miltiorrhiza* Bunge roots using a Soxhlet system. In this method, 10 g of root material was placed into a 250-mL extractor and continuously refluxed with 100 mL of boiling ethanol. The extraction yield of all tanshinones increased as the extraction time was extended from 30 to 90 min. However, when the extraction time exceeded 90 min, a notable decline in extraction efficiency was recorded (Pan et al. 2002; Fikri et al. 2022).

#### Microwave-assisted extraction (MAE)

Tanshinone I, tanshinone IIA, and cryptotanshinone were isolated from *S. miltiorrhiza* Bunge using a household microwave oven that was modified by adding a water condenser, magnetic stirrer, time-control system, and temperature sensor. Ten grams of root material was suspended in 100 mL of ethanol and subjected to programmed irradiation cycles consisting of 25 s of heating until the temperature reached 80 °C, followed by short cooling (10 min) heating pulses to prevent super-boiling. The maximum extraction yield was obtained after 2 min of irradiation. Prolonged exposure beyond 2 min of irradiation led to reduced yields due to the thermal and photo-oxidative degradation of tanshinones. Therefore, a 2-min duration was determined to be optimal (Pan et al. 2002). MAE of tanshinones can be optimized by balancing pre-treatment duration and solvent–solid ratio. A 45-min pre-leaching at room-temperature step before a 2-min MAE cycle significantly enhanced tanshinone extraction. However, extending the pre-leaching period over 45 min resulted in reduced yields. Furthermore, a liquid-to-solid ratio up to 10:1 (mL/g) demonstrated maximal extraction efficiency, minimizing energy consumption (Pan et al. 2001).

#### Ionic liquid–based ultrahigh pressure-assisted extraction (IL-UPE)

Optimization conditions for IL-UPE revealed that a solvent system containing 0.5 M 1-octyl-3-methylimidazolium hexafluorophosphate in ethanol, combined with 200 MPa pressure, a solid-to-liquid ratio of 1:20 (g/mL), and a 2-min extraction time, produced the highest extraction efficiency of tanshinone I, tanshinone IIA, dihydrotanshinone, miltirone, and cryptotanshinone from 0.5 g of *S. miltiorrhiza* Bunge roots (Liu et al. 2013).

#### Supercritical fluid extraction (SFE)

SFE utilizes CO_2_ above its critical pressure and temperature to achieve highly efficient and selective extraction of bioactive compounds. It was optimized using a CO_2_ flow rate of 3.23 L/min, 38.50 MPa pressure, and 52.21% peanut oil as a co-solvent for 15 min. These parameters enabled efficient extraction of dihydrotanshinone, cryptotanshinone, tanshinone I, and tanshinone IIA from *S.miltiorrhiza* (Danshen) (Fikri et al. 2022).

#### High-speed counter-current chromatography (HSCCC)

 Seven tanshinones including tanshinone I, tanshinone IIA, dihydrotanshinone I, neo-przewaquinone A cryptotanshinone, miltirone, and 1,2,15,16-tetrahydrotanshiquinone were isolated from *S. miltiorrhiza* Bunge using reflux extraction with ethyl acetate, followed by HSCCC purification. HSCCC employed light petroleum–ethyl acetate–methanol–water (6:4:6.5:3.5, v/v), where the lower phase served as the mobile phase and the upper phase as the stationary phase (Sun et al. 2011a). An alternative HSCCC strategy utilized two-sequential solvent systems, A ((10:5.5:4.5, v/v) *n*-hexane–ethanol–water) and B ((10:7:3, v/v) *n*-hexane–ethanol–water), that effectively separated six compounds including tanshinone I, tanshinone IIA, methylenetanshiquinone, dihydrotanshinone I, cryptotanshinone, and danshenxinkun B (Fikri et al. 2022).

## Pharmacological activities

### Effect of tanshinones as anti-inflammatory and antioxidants

Phytochemical investigations have revealed that the pharmacological properties of tanshinones are attributable to two major classes of constituents: hydrophilic phenolic acids and lipophilic abietane-type diterpene quinones, the latter collectively termed tanshinones. The major tanshinones include tanshinone IIA, cryptotanshinone, tanshinone I, and dihydrotanshinone I, each exhibiting distinct yet overlapping bioactivities (Wang et al. 2007). Of particular significance is their dual capacity to suppress pro-inflammatory cascades while activating endogenous antioxidant defenses a combination particularly relevant to disorders characterized by chronic inflammation and oxidative stress (Fang et al. 2021). Accumulating evidence indicates that tanshinone IIA and cryptotanshinone suppress pro-inflammatory cytokine production in immune and metabolic cell types. In human adipocytes stimulated with TNF-α, these compounds significantly downregulated IL-6, IL-8, MCP-1, and CXCL1 expression through inhibition of NF-κB nuclear translocation and modulation of inflammation-associated microRNAs (Carpi et al. 2023b). The NF-κB pathway represents a central target of tanshinone-mediated anti-inflammatory effects, with tanshinone IIA preventing phosphorylation and degradation of inhibitor κBα (IκBα), thereby retaining NF-κB dimers in the cytoplasm and preventing transcription of pro-inflammatory target genes (Ren et al. 2019). Parallel findings in macrophages demonstrated that tanshinone I disrupts NLRP3 inflammasome assembly by preventing NLRP3–ASC interaction, resulting in reduced caspase-1 activation and IL-1β maturation (Zhao et al. 2023). This inflammasome-inhibitory activity has been further corroborated in studies of sodium tanshinone IIA sulfonate (STS), which suppressed NLRP3 phosphorylation at serine 194, inhibited NLRP3 dimer formation, and reduced NLRP3-PYCARD (ASC) colocalization in endothelial cells and vascular smooth muscle cells under hyperglycemic conditions. These molecular effects translated into in vivo protection against endotoxemia and diet-induced steatohepatitis, indicating suppression of systemic inflammation. (Wei et al. 2024). The anti-inflammatory actions of tanshinones extend beyond NF-κB and inflammasome modulation to encompass inhibition of mitogen-activated protein kinase (MAPK) cascades. Tanshinone IIA suppresses phosphorylation of extracellular signal-regulated kinase 1/2 (ERK1/2), p38 MAPK, and c-Jun N-terminal kinase (JNK), thereby reducing activator protein-1 (AP-1)-mediated transcription of inflammatory mediators (Xu et al. 2015). Additionally, cryptotanshinone has been shown to inhibit signal transducer and activator of transcription 3 (STAT3) phosphorylation, further contributing to the attenuation of inflammatory gene expression (Xu et al. 2015).

Beyond cytokine regulation, tanshinones exert marked antioxidant effects by activating Nrf2-dependent transcription of phase II detoxifying enzymes as heme oxygenase-1, superoxide dismutase, and glutathione peroxidase, while reducing mitochondrial ROS generation and NADPH oxidase activity (Ding et al. 2025b). Dihydrotanshinone I has further been shown to protect endothelial cells from oxidative injury by stabilizing mitochondrial membrane potential and attenuating lipid peroxidation (Li et al. 2025a). Together, these findings establish tanshinones as upstream modulators of inflammatory amplification loops driven by oxidative stress, a hallmark of metabolic, cardiovascular, and immune-mediated disorders (Li et al. 2025a). Tanshinone IIA promotes Nrf2 nuclear translocation and transcriptional activation of heme oxygenase-1 (HO-1), NAD(P)H:quinone oxidoreductase 1 (NQO1), superoxide dismutase (SOD), and glutathione peroxidase (GPx), while simultaneously reducing mitochondrial reactive oxygen species (ROS) generation and NADPH oxidase activity (Tao et al. 2013b). This mitochondrial protective effect is particularly significant given the central role of mitochondrial dysfunction in driving oxidative stress and inflammatory signaling in chronic diseases (Yang et al. 2020).The Nrf2 activation mechanism involves modification of Kelch-like ECH-associated protein 1 (Keap1) cysteine residues, leading to conformational changes that release Nrf2 for nuclear translocation (Itoh et al. 2010).

**Fig. 2:**
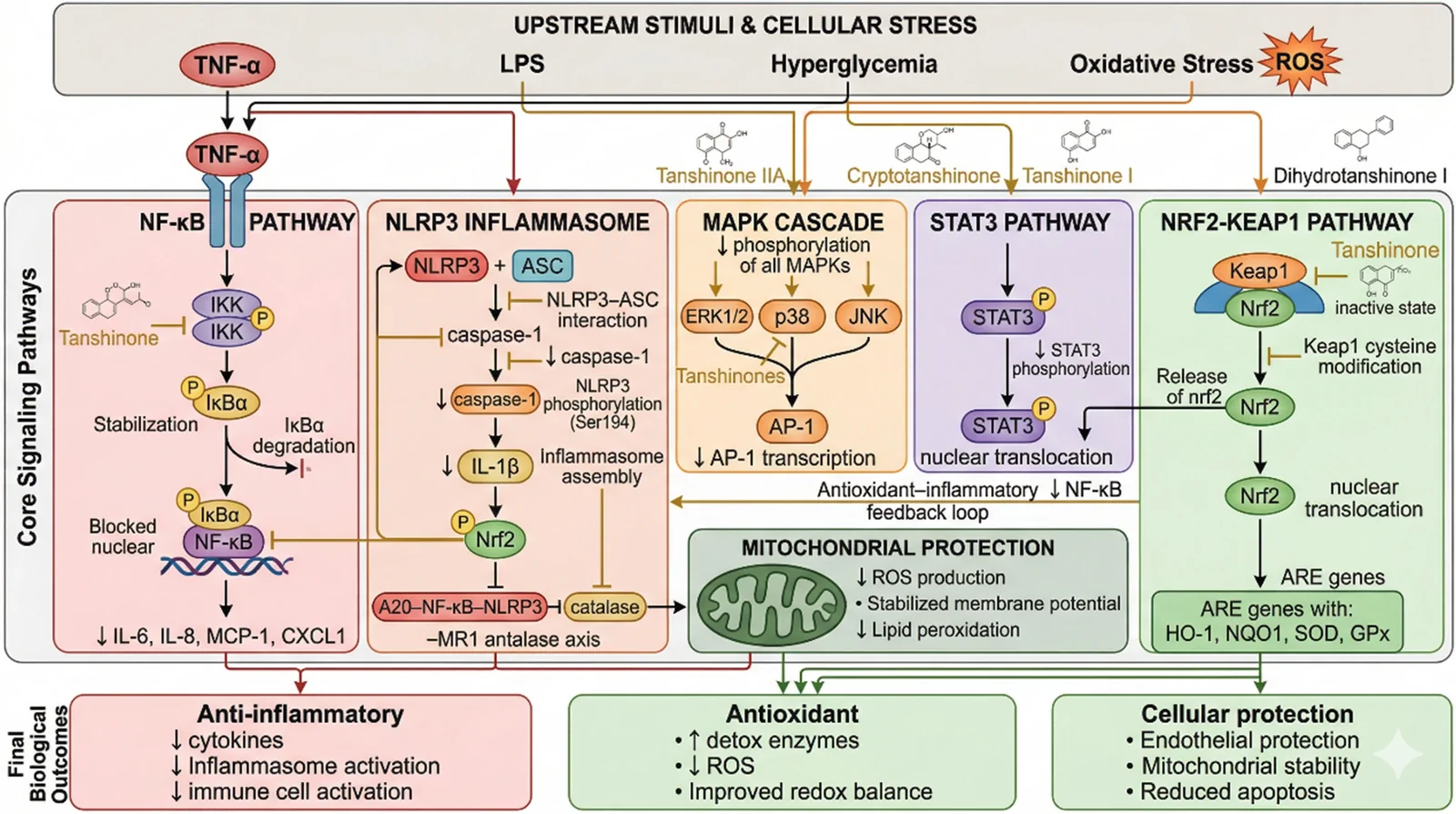
Anti-inflammatory and antioxidant mechanisms of tanshinones. Schematic illustrating the dual modulatory actions of major tanshinones (tanshinone IIA, cryptotanshinone, tanshinone I, dihydrotanshinone I). The left panel depicts anti-inflammatory effects via inhibition of NF-κB signaling (IκBα stabilization), suppression of pro-inflammatory cytokines (IL-6, IL-8, TNF-α), inhibition of MAPK (ERK, p38, JNK) and STAT3 pathways, and disruption of NLRP3 inflammasome assembly. The right panel highlights antioxidant mechanisms through activation of the Nrf2/ARE pathway, promoting expression of HO-1, NQO1, SOD, and GPx, alongside reduction of mitochondrial ROS and NADPH oxidase activity. Central crosstalk illustrates Nrf2-mediated suppression of NF-κB and integration of A20–NF-κB–NLRP3 signaling, collectively attenuating oxidative stress–driven inflammatory amplification

Recent investigations have revealed crosstalk between tanshinone-mediated antioxidant and anti-inflammatory pathways. The Nrf2 ARE pathway negatively regulates NF-κB transcriptional activity, creating a feedback loop wherein tanshinone-induced antioxidant gene expression contributes to suppression of inflammatory mediators (Wakabayashi et al. 2010). Furthermore, tanshinone IIA has been shown to modulate the A20-NFκB-NLRP3 inflammasome-catalase pathway, linking inflammasome inhibition with preservation of antioxidant enzyme activity (Zhang et al. 2025a). Together, these findings establish tanshinones as upstream modulators of inflammatory amplification loops driven by oxidative stress, a hallmark of metabolic, cardiovascular, and immune-mediated disorders, as shown in Table S1 and Fig. 2.

## Effect of tanshinones on respiratory system disorders

### Molecular and cellular signaling networks

At the molecular level, the therapeutic efficacy of tanshinones across diverse pulmonary pathologies is orchestrated through a highly coordinated, multi-targeted signaling network. A central axis of this protective mechanism is the potent suppression of pro-inflammatory transcription factors, primarily the NF-κB and signal transducer and activator of transcription 6 (STAT6) pathways (Li et al. 2020a). In conditions characterized by T-helper 2 (Th2)-dominant immune profiles, tanshinones directly inhibit the phosphorylation and activation of STAT6, effectively putting a molecular brake on downstream genetic networks that govern mucus hypersecretion and allergic inflammation. Concurrently, tanshinones interfere with the MAPK cascades, actively suppressing the phosphorylation of ERK1/2 and p38 MAPK to downregulate downstream systemic inflammatory amplification loops (Tian et al. 2022). Complementing this anti-inflammatory signaling checkpoint is the simultaneous, structural activation of endogenous antioxidant defenses. Tanshinones directly stimulate the nuclear factor erythroid 2-related factor 2 (Nrf2)/Keap1 ARE pathway. This cellular activation drives the nuclear translocation of Nrf2, promoting the transcription of essential phase II detoxifying and antioxidant enzymes while preserving cellular and mitochondrial structural integrity in alveolar macrophages (Ding et al. 2025b). Furthermore, in the epithelial boundary layer, this molecular network preserves architectural barrier integrity by maintaining tight junction protein expressions and directly minimizing ROS-dependent epithelial permeability (Zhang et al. 2024a).

#### Preclinical in vivo validation

The structural execution of these molecular pathways across different pulmonary pathologies has been systematically consolidated into a high-density preclinical matrix. As detailed in Table 1, these distinct in vivo and in vitro configurations map out the precise phenotypic changes, tissue-protective outcomes, and downstream master signaling cascades such as the Nrf2-Keap1, STAT6, and TLR4/NF-κB axes that are targeted by specific naturally occurring abietane diterpene monomers (Table 1 and Figure S1).

**Table 1:** Preclinical airway models, molecular mechanisms, and therapeutic outcomes of tanshinones

Pulmonary pathology	Preclinical in vivo model	Active compound/derivative	Phenotypic and tissue-protective outcomes	Key molecular and cellular mechanisms	References
Asthma and allergic airway inflammation	Ovalbumin-induced murine asthma	Tanshinone IIA	Reduced airway hyperresponsiveness Decreased eosinophilic tissue infiltration Suppressed goblet cell hyperplasia	Systematic downregulation of classic Th2 cytokines (IL-4, IL-5, IL-13) Enhanced pulmonary Nrf2 activation Diminished tissue levels of malondialdehyde	Ding et al. 2025b
Asthma and allergic airway inflammation	Airway epithelial cell/smooth muscle culture	Cryptotanshinone	Inhibited mucus hypersecretion	Suppression of STAT6 phosphorylation Downregulation of downstream MUC5AC expression	Li et al. 2020a
Chronic obstructive pulmonary disease (COPD)	Cigarette smoke– and lipopolysaccharide (LPS)–induced rodent COPD	Sodium tanshinone IIA sulfonate (water-soluble derivative)	Attenuated severe emphysematous changes Reduced tissue neutrophil accumulation Preserved alveolar architecture and global lung function	Inhibition of ERK1/2 and p38 MAPK pathways Capped production of pro-inflammatory cytokines (IL-6 and TNF-α)	Tian et al. 2022
Chronic obstructive pulmonary disease (COPD)	Cigarette smoke- and LPS-exposed alveolar macrophages	Cryptotanshinone	Preserved mitochondrial structural integrity Alleviated macrophage-driven pathology	Activation of the Keap1/Nrf2 antioxidant signaling axis Direct reduction of ROS-dependent inflammatory injury	Zhang et al. 2024a
Chronic obstructive pulmonary disease (COPD)	Cigarette smoke-exposed airway epithelium	Tanshinone IIA	Protected against epithelial barrier disruption Reduced total epithelial macromolecular permeability	Maintenance of tight junction protein expression patterns	Yang et al. 2023b
Acute lung injury (ALI)	High-dose endotoxin- or LPS-induced rodent ALI	Tanshinone IIA	Rapidly limited acute pulmonary edema Blocked widespread inflammatory cell infiltration	Targeted inhibition of upstream TLR4/NF-κB signaling clusters Simultaneous activation of Nrf2-mediated antioxidant defenses	Xu et al. 2011
Cancer-associated pulmonary inflammation	Preclinical lung tumor models	Tanshinone-rich extracts	Suppressed inflammatory cell infiltration Inhibited tumor angiogenesis Suggested potential chemopreventive phenotypes	Strategic suppression of inflammation-driven carcinogenesis Active modulation of the inflammatory tumor microenvironment	Xu et al. 2024

#### Translational roadblocks and clinical horizons

While these preclinical findings provide a solid functional foundation, several distinct translational complexities must be addressed before moving toward human clinical protocols. A primary metabolic bottleneck is the context-dependent cellular targeting of these abietane diterpenes, as seen by the requirement for stable drug exposure within alveolar macrophages to treat chronic diseases like COPD. Unformulated crystalline fractions suffer from poor regional bioavailability, poor aqueous solubility, and rapid phase II metabolic clearance when delivered systemically. To shift these clinical horizons, advanced formulation strategies are focusing on local, non-invasive direct pulmonary delivery systems. Utilizing inhalable polymeric nanocarriers, liposomes, or specialized lipid nanocapsules can optimize aerosol transport parameters, facilitate penetration through thick mucosal barriers, and sustain drug release directly at the alveolar-capillary junction (Wani 2021). Furthermore, the emerging observation that tanshinone-rich extracts can suppress angiogenesis and inflammatory cell infiltration within lung tumor microenvironments introduces an intriguing secondary clinical application, highlighting the potential use of these multi-targeted monomers as chemopreventive adjuncts to suppress inflammation-driven pulmonary carcinogenesis.

### Effect of tanshinones on gynecological disorders

#### Molecular and cellular signaling networks

At the molecular level, the therapeutic efficacy of tanshinones across various gynecological disorders is orchestrated through a highly integrated regulatory network that bridges hormonal, inflammatory, and metabolic signaling paths. A primary mechanism underlying their anti-endometriotic and anti-tumor action is the potent inhibition of the phosphatidylinositol 3-kinase/protein kinase B/mammalian target of rapamycin (PI3K/Akt/mTOR) signaling axis, which suppresses downstream matrices governing cell proliferation, tissue invasion, and extracellular matrix remodeling. Concurrently, tanshinones alter the microvascular environment by downregulating key pro-angiogenic factors, including vascular endothelial growth factor (VEGF), matrix metalloproteinase-9 (MMP-9) (Zhao et al. 2026), and hypoxia-inducible factor-1α (HIF-1α) (Jin et al. 2022), effectively starving lesions and tumor masses of vital neovascularization networks. At the cellular level, these abietane diterpenes suppress epithelial–mesenchymal transition (EMT) to inhibit cellular migration, while systematically activating apoptotic cascades through the modulation of internal pro-apoptotic Bcl-2-associated X protein/B-cell lymphoma 2 (Bax/Bcl-2) ratios (Yu et al. 2025), the activation of caspase-3, the upregulation of p53-dependent pathways, and the core suppression of silent information regulator 1 (SIRT1) (Zou et al. 2023) and STAT3 survival signaling (Li et al. 2015). Furthermore, protective and homeostatic effects within ovarian tissues are mediated via the activation of the Hippo signaling pathway alongside the coordinated balancing of protective protein kinase cascades (Figure S2).

#### Preclinical in vivo validation

The structural execution of these molecular pathways across different gynecological pathologies has been systematically consolidated into a high-density preclinical matrix. As detailed in Table 2, these distinct in vivo and in vitro configurations map out the precise phenotypic changes, tissue-protective outcomes, and downstream master signaling cascades such as the PI3K/Akt/mTOR, Hippo, and STAT3 axes that are targeted by specific naturally occurring abietane diterpene monomers (Table 2).

**Table 2:** Preclinical gynecological models, molecular mechanisms, and therapeutic outcomes of tanshinones

Gynecological pathology	Preclinical in vivo/in vitro model	Active compound/derivative	Phenotypic and tissue-protective outcomes	Key molecular and cellular mechanisms	References
Endometriosis	Surgically induced endometrial rat model	Tanshinone IIA	Significant reduction in ectopic lesion volume and total weight Regression of established lesions	Lowering of localized peritoneal concentrations of inflammatory drivers (tumor necrosis factor alpha; TNF-α, IL-1β, PGE2)	Zhao et al. 2026
Endometriosis	Ectopic endometrial cell culture and invasion models	Tanshinone IIA	Suppression of tissue invasion and extracellular matrix remodeling Restriction of cellular migratory and invasive capacity	Inhibition of PI3K/Akt/mTOR signaling Downregulation of matrix metalloproteinase-9 (MMP-9) and VEGF Suppression of epithelial–mesenchymal transition (EMT)	Zhang et al. 2023a
Endometriosis	Ectopic endometrial lesion neovascularization models	Tanshinone IIA	Reduced microvessel density within endometriotic lesions Blockade of neovascularization cascades required to maintain ectopic tissues	Decreased expression of pro-angiogenic factors (VEGF and HIF-1α)	Jin et al. 2022
Endometriosis	Ectopic endometrial cell apoptosis models	Tanshinone IIA	Promotion of programmed cell death (apoptosis) in ectopic cells Prevention of disease progression	Modulation of Bax/Bcl-2 ratios Activation of cleavage enzyme Caspase-3	Yu et al. 2025
Toxic ovarian damage	Tri-*ortho*-cresyl phosphate (TOCP)-induced mouse model	Tanshinone IIA	Amelioration of chemical-induced ovarian dysfunction Structural protection of the ovarian reserve from environmental toxicants	Coordinated activation of the Hippo signaling axis Concurrent balancing of AKT/MAPK pathway activities	Ma et al. 2025
Endometrial carcinoma	Endometrial carcinoma cell line assays	Tanshinone IIA and neo-tanshinlactone derivatives	Induction of robust growth arrest Stimulation of cell death in malignant tissues	Activation of p53-dependent apoptotic pathways Inhibition of SIRT1-mediated cellular survival signaling	Zou et al. 2023
Ovarian cancer	Ovarian cancer cell proliferation models	Tanshinone IIA	Inhibition of uninhibited cell proliferation Triggering of programmed cell death	Targeted modulation of STAT3 signaling loops Strategic generation of intracellular reactive oxygen species (ROS)	Li et al. 2018
Breast cancer	Hormone-responsive breast cancer models	Cryptotanshinone	Potent anti-proliferative actions in hormone-dependent tumors	Post-transcriptional bottlenecking via active suppression of classical estrogen receptor signaling	Li et al. 2015

### Translational roadblocks and clinical horizons

Translating these compelling multi-system preclinical findings into clinical settings requires addressing several explicit physiological complexities, particularly regarding polycystic ovary syndrome (PCOS) and associated metabolic dysfunctions. PCOS represents a highly complex endocrinopathy driven by a combination of hyperandrogenism, ovulatory failure, peripheral insulin resistance, and chronic low-grade systemic inflammation. While direct clinical trials focusing exclusively on isolated, purified tanshinone monomers remain limited in this domain, related clinical evidence utilizing whole-herb *Salvia officinalis* extracts in women with PCOS has demonstrated promising improvements in overall insulin sensitivity, glucose handling, and serum hormonal parameters (Maleki-Hajiagha et al. 2025). The robust anti-inflammatory, antioxidant, and endocrine-modulating characteristics of pure tanshinone monomers position them as high-priority candidate agents to resolve the chronic oxidative stress and macrophage-driven inflammatory microenvironment that characterize human PCOS pathogenesis (Jin et al. 2019). However, a significant translational boundary remains their lipophilic chemistry, which restricts aqueous solubility and clinical bioavailability. Overcoming these metabolic constraints will depend upon developing advanced nanomedicine platforms such as target-modified liposomes, solid lipid nanoparticles, or nanoemulsions to protect the diterpenoid structure from rapid first-pass clearance, extend total plasma residence time, and ensure precise, ligand-mediated delivery to target reproductive and metabolic tissues.

### Anti-cancer and immunomodulatory effects of tanshinones

#### Molecular and cellular signaling networks

At the molecular level, the comprehensive antitumor and immunomodulatory profiles of tanshinone monomers specifically tanshinone I, tanshinone IIA, cryptotanshinone, and dihydrotanshinone I are driven by their ability to simultaneously target multiple conserved oncogenic signaling networks. A primary mechanism involves the induction of apoptosis mediated by severe endoplasmic reticulum (ER) stress through the activation of the unfolded protein response (UPR) pathway. This molecular cascade triggers three major transmembrane sensors: protein kinase RNA-like endoplasmic reticulum kinase (PERK), inositol-requiring enzyme 1 alpha (IRE1α), and activating transcription factor 6 (ATF6). Activation of these sensors drives the upregulation of pro-apoptotic downstream mediators, notably C/EBP homologous protein (CHOP) and the caspase cascade, leading directly to irreversible mitochondrial dysfunction and cell death (Yan et al. 2012; Chiu And Su 2017). Concurrently, tanshinones alter the expressions of the Bcl-2 family, systematically increasing the ratio of Bax relative to anti-apoptotic Bcl-2.

Beyond apoptosis, tanshinones halt cell-cycle progression and suppress tumor cell proliferation by blocking key upstream growth-regulatory nodes, including the PI3K/Akt/mTOR and MAPK pathways, while simultaneously activating the AMP-activated protein kinase (AMPK) energy-sensing network. This dual action allows tanshinones to enhance autophagic flux, upregulating key autophagy-related proteins such as beclin-1 and microtubule-associated protein 1 light chain 3-II (LC3-II) to degrade survival factors within the tumor cell. Within the tumor microenvironment, these monomers serve as post-transcriptional bottlenecks that disrupt the STAT3 and Janus kinase (JAK/STAT3) axes. This downregulates crucial downstream oncogenic effectors such as survivin, cyclin D1, and B-cell lymphoma-extra-large (Bcl-XL). Furthermore, certain monomers drive cell death by stimulating intracellular ROS generation and p38 MAPK signaling, which induces severe mitochondrial stress in malignant cells (Lv et al. 2018; Liao et al. 2019) (Figure S3).

### Preclinical in vivo validation

The structural execution of these molecular pathways across different cancer types has been systematically consolidated into a high-density preclinical matrix. As detailed in Table 3, these distinct in vivo and in vitro configurations map out the precise phenotypic changes, tissue-protective outcomes, and downstream master signaling cascades targeted by specific naturally occurring abietane diterpene monomers.

**Table 3:** Preclinical malignancy models, molecular mechanisms, and therapeutic outcomes of tanshinones

Oncological target	Preclinical in vivo/in vitro model	Active compound/derivative	Phenotypic and antitumor outcomes	Key molecular and cellular mechanisms	References
Pancreatic and breast cancer	Human pancreatic cancer (BxPC-3) and breast cancer (BT-20) cells	Tanshinone IIA	Decreased tumor cell viability Induction of caspase-dependent apoptosis	Induction of endoplasmic reticulum (ER) stress Activation of unfolded protein response (UPR) pathways (PERK, IRE1α, ATF6) Downstream activation of pro-apoptotic CHOP and caspases	Yan et al. 2012; Chiu And Su 2017
Breast and lung cancer	Breast cancer (MCF-7) cells and non-small-cell lung cancer (NSCLC) models	Tanshinone IIA	Suppression of cell survival Induction of cell-cycle arrest and inhibition of proliferation	Attenuation of aberrant PI3K/Akt/mTOR and MAPK signaling Upregulation of AMPK metabolic regulatory activity	Lv et al. 2018; Liao et al. 2019
Tumor invasion and metastasis	Multiple in vivo malignancy models (leukemia, HCC, breast, lung, colorectal, etc.)	Tanshinone IIA	Significant reduction in the migratory and invasive capacities of cancer cells in vivo Restriction of systemic metastatic progression	Downregulation of matrix metalloproteinases (MMP-2 and MMP-9) Blockade of extracellular matrix degradation Regulation of epithelial–mesenchymal transition (EMT) pathways	Yu et al. 2017; Zhang et al. 2019
Tumor angiogenesis	Endothelial progenitor cell and colorectal cancer models	Tanshinone IIA	Inhibition of VEGF-induced endothelial migration and capillary tube formation Blockade of tumor vascularization under normoxic and hypoxic conditions	Suppression of downstream PLC/Akt and JNK pathways Disruption of β-catenin-mediated angiogenic signaling	Lee et al. 2017; Sui et al. 2017
Tumor immunosuppression	Macrophage, dendritic, and natural killer (NK) cell models	Cryptotanshinone	Repression of immune suppression within the tumor microenvironment Promotion of tumor-associated macrophage polarization to M1 phenotype Enhanced dendritic cell maturation and antigen presentation Enhanced NK cell differentiation and cytotoxic activity	Blockade of aberrant STAT3 signaling networks Downregulation of oncogenic effectors (survivin, cyclin D1, Bcl-XL) Activation of the p38 mitogen-activated protein kinase (p38 MAPK) pathway	Shin et al. 2009; Kim et al. 2012; Ke et al. 2017; Han et al. 2019; Liu et al. 2019
Immune checkpoint blockade	Non-small-cell lung cancer (NSCLC) models	Tanshinone IIA	Enhanced therapeutic efficacy of anti-PD-1 immunotherapy in vivo Improved baseline antitumor immune responses	Repression of programmed death-ligand 1 (PD-L1) expression ER stress-mediated activation of JNK signaling pathways	Zhang et al. 2024b
Chemoresistance	Resistant tumor cell models	Tanshinone monomers	Reversal of acquired chemoresistance Sensitization of tumor cells to conventional cytotoxic agents (doxorubicin, cisplatin)	Synergistic interaction with conventional chemotherapeutic mechanisms	Liao et al. 2019; Li et al. 2021
Metastasis and apoptosis	Comprehensive cancer cell line panels	Tanshinone I	Suppression of tumor metastasis Induction of programmed cell death	Modulation of the upstream JAK/STAT3 signaling axis Negative regulation of MMP-2 and MMP-9 abundance	Wang et al. 2019b
Mitochondrial cytotoxicity	Diverse cancer cell configurations	Dihydrotanshinone I	Potent direct cytotoxicity Activation of mitochondrial apoptosis	Rapid intracellular accumulation of reactive oxygen species (ROS) Downstream activation of the p38 MAPK signaling cascade Induction of mitochondrial outer membrane permeabilization	Lee et al. 2009; Wang et al. 2013

#### Translational roadblocks and clinical horizons

Despite the extensive preclinical footprint and clear therapeutic value demonstrated by these monomers, transitioning them into human oncological care presents significant translational boundaries. The overwhelming majority of current evidence is limited to in vitro assays and animal tumor models; thus, further rigorous pharmacological investigation and well-designed human clinical trials are mandatory to confirm safety, efficacy, and real-world pharmacokinetics. The primary translational bottleneck stems from the lipophilic abietane core, which causes poor aqueous solubility, low systemic bioavailability, and rapid first-pass phase II hepatic glucuronidation. These limitations often prevent the compounds from reaching required therapeutic concentrations within the complex tumor microenvironment when administered orally in traditional unformulated designs.

To expand these clinical horizons, current research is focusing on innovative nanomedicine and targeted drug delivery systems. Formulating tanshinones into advanced nanocarriers such as polymer-conjugated nanoparticles, solid lipid nanoparticles, nanoemulsions, or target-functionalized liposomes can shield the active diterpenoid structure from rapid metabolic clearance and extend total plasma circulation time. These nanotechnological platforms can be surface-modified with specific ligands to exploit the enhanced permeability and retention (EPR) effect or directly target overexpressed receptors on malignant cells. This approach allows for the accumulation of active compounds directly within the tumor parenchyma while sparing healthy tissues from off-target toxicities. Moving forward, the clinical development of tanshinones will likely favor their application not as isolated monotherapies, but as multi-targeted adjuncts or sensitizing agents. When strategically paired with conventional chemotherapeutic regimens or immune checkpoint inhibitors, they can help bypass drug resistance pathways, lower mandatory toxic drug doses, and enhance overall clinical outcomes across multiple aggressive malignancies.

#### Protective effects of tanshinones against drug-induced toxicity

##### Molecular and cellular signaling networks

At the molecular level, the organ-protective action of tanshinone monomers against drug-induced toxicities is driven by a highly coordinated, multi-system network that controls oxidative stress, xenobiotic detoxification, and fibrotic tissue remodeling. A foundational mechanism across multiple organs is the direct activation of the Nrf2 signaling pathway (Jiang et al. 2022). Upon stimulation by tanshinones, Nrf2 dissociates from Keap1 and undergoes nuclear translocation, binding to antioxidant response elements to drive the transcription of essential Phase II detoxifying and antioxidant enzymes. This molecular network significantly boosts the expression of glutamate-cysteine ligase catalytic subunit (GCLC), NQO1, and HO-1, while simultaneously restoring vital endogenous antioxidant pools. Mechanistically, this transcriptional loop is frequently controlled via the long non-coding RNA HOX transcript antisense intergenic RNA (HOTAIR)–Nrf2 network axis, which subsequently upregulates multi-specific efflux pumps (Zhang et al. 2020).

Concurrently, tanshinones alter anti-apoptotic signaling pathways to shield cells from drug-induced mitochondrial collapse. They upregulate the PI3K/Akt survival cascade to decrease intracellular ROS accumulation, increase expression of anti-apoptotic Bcl-XL, prevent the mitochondrial release of cytochrome *c*, and block the downstream activation of the cleavage enzyme caspase-3 (Hong et al. 2012). In chronic toxicity models, these abietane diterpenes serve as post-transcriptional bottlenecks that disrupt the transforming growth factor-β1 (TGF-β1)/Smad3 and Smad2/3 signaling pathways. By operating via sirtuin 3 (SIRT3)-dependent and epigenetic transcriptional mechanisms, tanshinones repress Smad phosphorylation, prevent EMT, restore critical epithelial architectural markers like E-cadherin, and systematically suppress downstream extracellular matrix deposition and fibrotic tissue remodeling (Figure S4).

##### Preclinical in vivo validation

The structural execution of these molecular pathways across different models of drug-induced toxicity has been systematically consolidated into a high-density preclinical matrix. As detailed in Table 4, these distinct in vivo and in vitro configurations map out the precise phenotypic changes, tissue-protective outcomes, and downstream master signaling cascades such as the Nrf2-Keap1, PI3K/Akt, and TGF-β1/Smad3 axes that are targeted by specific naturally occurring abietane diterpene monomers.

**Table 4:** Preclinical toxicity models, molecular mechanisms, and protective outcomes of tanshinones

Target toxicity type	Preclinical in vivo/in vitro model	Active compound/derivative	Phenotypic and tissue-protective outcomes	Key molecular and cellular mechanisms	References
Acute hepatotoxicity	Acetaminophen (APAP) overdose rodent models	Tanshinone IIA	Marked attenuation of hepatic injury Prevention of severe lipid peroxidation and acute hepatocellular damage	Activation of Nrf2 signaling pathway to upregulate GCLC, NQO1, and HO-1 Restoration of native antioxidant pools (GSH, SOD, CAT, GPx)	Li et al. 2020b; Wang et al. 2016
Xenobiotic liver clearance	APAP metabolite detoxification models	Tanshinone IIA	Enhanced detoxification and systemic elimination of toxic reactive NAPQI Reduced toxic tissue accumulation	Upregulation of apical efflux transporters MRP2 and MRP4 via Nrf2 transcriptional activation Regulation of the long non-coding RNA HOTAIR–Nrf2–MRP2/4 axis	Zhang et al. 2020
Drug-induced nephrotoxicity	APAP-induced acute kidney injury	Tanshinone IIA	Successful alleviation of acute tubular necrosis	Activation of the renal Nrf2–MRP2/4 axis to accelerate clearance of NAPQI in renal cells	Zhang et al. 2022
Chemotherapy cardiotoxicity	Doxorubicin (DOX)-induced cardiomyopathy rodent models	Tanshinone I	Marked improvement in global cardiac function Preservation of cardiomyocyte mitochondrial structural integrity	Promotion of Nrf2 nuclear translocation and activation Upregulation of Nrf2-dependent antioxidant defense genes	Jiang et al. 2022
Chemotherapy cardiotoxicity	DOX-exposed cardiomyocyte culture	Tanshinone IIA	Attenuation of DOX-induced cardiomyocyte apoptosis Preservation of myocardial architectural integrity	Activation of the PI3K/Akt survival signaling cascade Suppression of mitochondrial cytochrome *c* release and caspase-3 cleavage Increased expression of anti-apoptotic Bcl-XL	Hong et al. 2012
Chemotherapy nephrotoxicity	DOX- or cisplatin-induced kidney injury models	Tanshinone IIA	Significant reduction in blood urea nitrogen (BUN) levels Improvement of mitochondrial function and suppression of ROS Attenuation of progressive renal fibrosis and collagen deposition	Inhibition of the profibrotic TGF-β1/Smad3 pathway via a SIRT3-dependent mechanism Direct post-transcriptional modulation of the solute carrier family 7 member 11 (SLC7A11) transporter	McSweeney et al. 2021; Dong et al. 2024; Fan et al. 2024; Qin et al. 2024; Jiang et al. 2025
Chemotherapy nephrotoxicity	Cisplatin-challenged renal models	Tanshinone I	Alleviation of acute cisplatin-induced nephrotoxicity Reduction of lipid peroxidation patterns	Enhancement of native antioxidant enzyme kinetics Successful restoration of cellular glutathione (GSH) levels in renal tissue	Wang et al. 2022b
Chemotherapy nephrotoxicity	Cisplatin-induced kidney injury models	Dihydrotanshinone I	Robust nephroprotection from heavy metal injury Suppression of apoptotic signaling cascades	Targeted modulation of transcriptional regulator tripartite motif-containing 28 (TRIM28) Regulation of the internal Bax/Bcl-2 equilibrium and inhibition of caspase-3	Sun et al. 2025
Drug-induced ototoxicity	Aminoglycoside (gentamicin)-induced hearing loss models	*Salvia miltiorrhiza* extracts	Distinctive attenuation of gentamicin-induced hearing impairment Protection and structural preservation of cochlear hair cells	Decreased expression of inducible nitric oxide synthase (iNOS) Reduced abundance of the apoptotic marker caspase-3	Wang et al. 2003; Shi et al. 2014
Peripheral neuropathy	Platinum agent (oxaliplatin)-induced CIPN models	Tanshinone IIA and cryptotanshinone	Dose-dependent attenuation of neuropathic pain and mechanical allodynia Preservation of sciatic nerve function and neuronal cell viability	Activation of the PI3K/Akt/mTOR pathway to stimulate protective autophagy Increased tissue nerve growth factor (NGF) levels Repression of calcitonin gene-related peptide (CGRP) expression	Xu et al. 2016; Di Cesare Mannelli et al. 2018; Cheng et al. 2019
Drug-induced lung fibrosis	Bleomycin-induced pulmonary fibrosis models	Tanshinone IIA	Limited pulmonary edema and inflammatory cell infiltration Attenuation of severe chronic fibrotic tissue remodeling	Suppression of inflammatory and fibrotic mediators (TNF-α, IL-1β, IL-6, cyclooxygenase-2 (COX-2), PGE2, iNOS, NO, MDA) Inhibition of TGF-β–induced EMT via suppression of Smad2/3 phosphorylation Maintenance of structural E-cadherin expression	He et al. 2015; Tang et al. 2015

### Translational roadblocks and clinical horizons

Transitioning these compelling organ-protective effects into human clinical protocols requires navigating several distinct pharmacological and metabolic boundaries. A primary translational challenge is balancing the cytoprotective effects of tanshinones in healthy tissues with the therapeutic goals of conventional chemotherapies. Because these monomers act as potent antioxidants and anti-apoptotic signaling modulators, future clinical trials must carefully verify that co-administration does not unintentionally shield malignant cells from the pro-oxidant, cytotoxic mechanisms of agents like doxorubicin or cisplatin. Additionally, the poor aqueous solubility and low oral bioavailability of the native abietane core limit the consistent attainment of therapeutic drug concentrations within targeted tissues like the myocardium, renal cortex, and peripheral nerves.

To expand these clinical horizons, current formulation research is turning toward advanced nanotechnological drug delivery systems. Encapsulating tanshinones into solid lipid nanoparticles, nanoemulsions, or ligand-functionalized liposomes protects the active diterpenoid structure from rapid first-pass phase II hepatic metabolism and extends its systemic circulation time. These advanced carriers can be surface-modified to target specific tissue compartments—such as utilizing mitochondrial-targeting moieties to deliver the compounds directly to cells experiencing severe drug-induced mitochondrial stress. Moving forward, designing smart, co-delivery nanoplatforms that combine chemotherapeutic agents with nanoformulated tanshinones could optimize treatment outcomes. This approach could enhance antitumor efficacy while minimizing toxic off-target damage to the heart, liver, and kidneys, presenting a viable path toward safer and more effective clinical oncology regimens.

### Effect of tanshinones on skeletal muscle disorders

#### Molecular and cellular signaling networks

At the molecular level, the pleiotropic protective action of tanshinone monomers in skeletal muscle disorders is driven by a highly integrated signaling grid that controls mitochondrial dynamics, fibro-adipogenic lineage commitment, and metabolic fuel utilization. A foundational mechanism involves preserving mitochondrial homeostasis and structural integrity. Tanshinones directly suppress the accumulation of mitochondrial reactive oxygen species (mtROS) and function as a post-transcriptional brake on excessive mitophagy and mitochondrial fission cascades, which limits downstream muscle protein degradation and prevents myotube atrophy (Han et al. 2024). To combat chronic tissue remodeling and injury-induced scarring, these abietane diterpenes target prominent profibrotic and pro-inflammatory nodes, specifically blocking the TGF-β1/Smad3 axis and downregulating the PI3K/Akt/COX-2 and intercellular adhesion molecule-1 (ICAM-1) signaling networks (Wang et al. 2006). Concurrently, they activate cellular regeneration programs by stimulating the proliferation and differentiation of muscle stem cells (MuSCs) via the dual activation of the MAPK and canonical PI3K/Akt survival pathways, driving the transcriptional upregulation of crucial myogenic regulatory factors.

Furthermore, tanshinones act as structural lineage switches within the injured muscle microenvironment. They suppress the aberrant fat accumulation and fatty infiltration that frequently destroys chronically damaged muscles by stimulating the canonical Wnt/β-catenin signaling pathway, which actively represses the adipogenic differentiation of resident fibro-adipogenic progenitors (Zhang et al. 2025b). On the metabolic front, these monomers reverse local endocrine defects by blocking systemic and tissue-specific renin–angiotensin systems, thereby lowering local angiotensin II production. This local hormonal blockade restores proper downstream insulin receptor substrate 1 (IRS1) signaling kinetics by preventing its inhibitory serine 307 phosphorylation. This molecular correction promotes the plasma membrane translocation of glucose transporter 4 (GLUT4), enhances functional cellular glucose uptake, and represses the advanced glycation end products/receptor for advanced glycation end products (AGEs/RAGE) pro-inflammatory signaling cascade to optimize overall cellular redox balance and metabolic efficiency during sustained muscular workload (Zhang et al. 2026) (Figure S5).

#### Preclinical in vivo validation

The structural execution of these molecular pathways across different skeletal muscle disorders has been systematically consolidated into a high-density preclinical matrix. As detailed in Table 5, these distinct in vivo and in vitro configurations map out the precise phenotypic changes, tissue-protective outcomes, and downstream master signaling cascades such as the Wnt/β-catenin, TGF-beta1/Smad3, and PI3K/Akt axes that are targeted by specific naturally occurring abietane diterpene monomers.

**Table 5:** Preclinical skeletal muscle models, molecular mechanisms, and therapeutic outcomes of tanshinones

Target myopathy/condition	Preclinical in vivo/in vitro model	Active compound/derivative	Phenotypic and tissue-protective outcomes	Key molecular and cellular mechanisms	References
Systemic inflammation and atrophy	Lipopolysaccharide (LPS)-induced rodent models	Tanshinone IIA	Mitigation of inflammation-induced skeletal muscle atrophy Restoration of total muscle mass, functional grip strength, and myofiber cross-sectional diameter	Prevention of mitochondrial ROS (mtROS) accumulation Inhibition of excessive mitophagy and mitochondrial fission cascades	Han et al. 2024
Ischemia–reperfusion (I/R) injury	Acute skeletal muscle I/R rodent injury models	Tanshinone IIA	Marked reduction of serum markers of muscle destruction (creatine phosphokinase, AST, LDH) Improved muscular tolerance to acute ischemic stress	Elevation of native tissue superoxide dismutase (SOD) activity Suppression of lipid peroxidation and localized apoptotic programs Direct inhibition of intercellular adhesion molecule-1 (ICAM-1)	Wang et al. 2006
Mechanical laceration and scarring	Traumatic skeletal muscle injury models	Sodium tanshinone IIA sulfonate (water-soluble derivative)	Potent anti-fibrotic actions Minimization of inflammatory cell infiltration and interstitial collagen deposition Acceleration of regenerative myotube development	Interruption of the profibrotic TGF-β1/Smad3 signaling network Suppression of the active PI3K/Akt/COX-2 axis Upregulation of core myogenic regulatory factors (MyoD, myosin heavy chain) in myoblasts	Ding et al. 2025a
Physical fatigue and exhaustion	Forced-swimming rodent endurance tests	Tanshinone IIA	Marked prolongation of total physical swimming performance time Significant enhancement of metabolic fuel utilization	Minimization of excessive post-exercise serum accumulation of glucose and lactate Optimization of structural metabolic efficiency during workload	Lin et al. 2017
Traumatic crush injury	Localized muscle crush rodent models	Tanshinone IIA	Accelerated functional recovery profiles Direct improvement of muscle force generation and myofiber repair	Activation of the mitogen-activated protein kinase (MAPK) signaling network Drive of muscle stem cell (MuSC) expansion and targeted differentiation	Liu et al. 2023
Orthopedic tendon–muscle tears	Chronic rotator cuff injury rodent models	Tanshinone IIA	Attenuation of severe degenerative fatty infiltration within the muscle belly Vastly superior long-term functional and biomechanical recovery	Activation of the canonical Wnt/β-catenin signaling pathway Suppression of the adipogenic lineage fate of fibro-adipogenic progenitors	Zhang et al. 2025b
Diabetic sarcopenia	Experimental diabetic muscle wasting configurations	Tanshinone IIA	Reversal of accelerated diabetic sarcopenia phenotype Successful restoration of native muscle mass, strength, and glucose clearance	Localized suppression of tissue renin activity and angiotensin II synthesis Restoration of proper insulin signaling via suppression of inhibitory IRS1/Ser307 phosphorylation Enhanced structural glucose transporter 4 (GLUT4) expression Attenuation of the AGEs/RAGE-mediated pro-inflammatory network	Zhang et al. 2026

#### Translational roadblocks and clinical horizons

Moving these highly promising multi-system preclinical findings into human therapeutic contexts requires navigating several distinct pharmacological and delivery boundaries. A primary translational bottleneck in treating skeletal muscle diseases is ensuring sufficient, stable drug accumulation across massive, diffuse, and frequently poorly vascularized muscle beds. Because native lipophilic tanshinones undergo rapid first-pass phase II hepatic metabolism and exhibit extremely poor aqueous solubility, achieving therapeutic concentrations in peripheral skeletal muscles *via *traditional oral delivery remains highly challenging. Furthermore, chronic conditions like diabetic sarcopenia or age-related muscle wasting require prolonged, stable treatment regimens, making traditional systemic therapies prone to off-target metabolic adjustments or variable systemic exposure over time.

To expand these clinical horizons, current formulation strategies are prioritizing the design of advanced, tissue-targeted nanotechnological platforms. Encapsulating tanshinones into muscle-homing polymeric nanoparticles, solid lipid nanoparticles, or surface-modified nanoemulsions can shield the abietane core from rapid enzymatic breakdown and drastically extend its systemic plasma half-life. These nanomedicines can be functionalized with specific targeting peptides that possess a high affinity for receptors overexpressed on regenerating muscle capillaries or expanding muscle stem cell niches. Additionally, for localized orthopedic or traumatic muscle injuries, such as rotator cuff tears or muscle lacerations, developing bio-absorbable, tanshinone-loaded nanofibrous scaffolds or injectable hydrogels represents a promising clinical direction. These localized delivery platforms can provide sustained, controlled release of the active monomer directly into the injury epicenter, maximizing anti-inflammatory, anti-fibrotic, and pro-myogenic regenerative activities exactly where tissue repair is needed, while completely avoiding systemic clearance bottlenecks.

#### Effect of tanshinones on cardiovascular diseases

##### Molecular and cellular signaling networks

At the molecular level, the cardioprotective and anti-remodeling actions of tanshinone monomers are driven by a highly integrated regulatory network that simultaneously controls ischemic cell death, extracellular matrix deposition, and metabolic survival signaling. A primary mechanism underlying tissue repair following ischemic insult is the direct upregulation of the master transcriptional regulators HIF-1α and VEGF, which drives microvascular neovascularization to rescue compromised myocardium. To arrest progressive ventricular remodeling and interstitial scarring, these abietane diterpenes modulate the delicate balance between MMPs and tissue inhibitors of metalloproteinases (TIMPs), successfully restoring collagen homeostasis and preserving baseline ventricular compliance (Wu et al. 2021).

Concurrently, tanshinones activate specific cell-survival signaling loops to block metabolic cardiomyocyte apoptosis in challenging microenvironments. They engage the membrane-bound kinin B2 receptor, which drives the downstream phosphorylation and activation of the protein kinase B (Akt) and glycogen synthase kinase-3β (GSK-3β) signaling pathways (Sun et al. 2011b). This explicit receptor-mediated cascade blocks pro-apoptotic signaling clusters and dampens local inflammatory pathways. Furthermore, when administered systemically or evaluated in clinical settings, water-soluble tanshinone derivatives exert potent systemic anti-inflammatory and lipid-modulating actions, significantly repressing high-sensitivity C-reactive protein (hs-CRP) synthesis and favorably resetting circulating lipid fractions by reducing total cholesterol, triglycerides, and low-density lipoprotein cholesterol (LDL-C) while elevating high-density lipoprotein cholesterol (HDL-C).

##### Preclinical in vivo validation

These molecular signaling mechanisms have been extensively validated across multiple well-established preclinical rodent models of acute myocardial infarction (MI), renovascular hypertension, and diabetes-induced heart failure. In rodent models of permanent coronary artery ligation or acute myocardial ischemia–reperfusion injury, administration of tanshinone IIA results in a distinct, measurable reduction in absolute infarct size and a substantial preservation of left ventricular systolic function, as reflected by increased left ventricular ejection fraction and improved global hemodynamic parameters (Zhu et al. 2023). This anatomical rescue is achieved through the active stimulation of local angiogenesis via the HIF-1α and VEGF cascades, demonstrating that tanshinone IIA facilitates structural myocardial repair beyond merely limiting acute necrosis (Zhu et al. 2023). Similarly, purified tanshinone I exhibits powerful cardioprotective activity in vivo, markedly attenuating acute ischemic injury parameters and maintaining overall myocardial structural integrity (Wu et al. 2021).

In long-term models of renovascular hypertension where interstitial scarring serves as a critical driver of progressive heart failure, treatment with tanshinone IIA significantly decreases severe myocardial fibrosis and balances out the localized MMP/TIMP ratio (Chen et al. 2021c). Notably, this anti-fibrotic tissue-protective profile occurs without significantly altering systemic blood pressure, proving that tanshinones exert a direct cardioprotective and collagen-stabilizing role independent of conventional antihypertensive hemodynamics (Chen et al. 2021c). Advanced combination strategies pairing tanshinone IIA with the natural isoflavone puerarin demonstrate further enhanced therapeutic synergy, showing superior suppression of inflammatory cell infiltration, reduced ventricular collagen deposition, and improved long-term ventricular geometry (spontaneous myocardial remodeling) (Miao et al. 2022). Furthermore, in streptozotocin-induced models of diabetic cardiomyopathy, tanshinone IIA reliably improves global cardiac function and suppresses cardiomyocyte apoptosis. This specific metabolic protection is mediated through the targeted activation of the kinin B2 receptor–Akt–GSK-3β signaling axis and the simultaneous attenuation of local inflammatory signaling; notably, the pharmacological blockade of the kinin B2 receptor completely abolishes these protective benefits, establishing explicit mechanistic dependency (Sun et al. 2011b).

#### Effect of tanshinones on kidney disorders

##### Molecular and cellular signaling networks

At the molecular level, the comprehensive nephroprotective action of tanshinone monomers is driven by a highly integrated regulatory signaling network that simultaneously controls extracellular matrix deposition, tissue inflammation, and cellular metabolic reprogramming. A foundational mechanism across multiple renal pathologies is the potent suppression of the TGF-β1 signaling axis, which operates as a master controller of progressive tissue scarring. Tanshinones function as post-transcriptional brakes on the downstream phosphorylation of Smad proteins and the activation of NF-κB transcription factors (Wang et al. 2015a; Nie and Li 2018). This combined action suppresses the transcriptional upregulation of crucial profibrotic effectors, including collagen IV and fibronectin, while simultaneously cutting off the production of pro-inflammatory cytokines within the renal parenchyma. Furthermore, specific monomers block the signaling of inhibin beta-A (INHBA), an important ligand belonging to the wider TGF-β superfamily, to provide additional protection against progressive structural modeling (Wu et al. 2022). Concurrently, tanshinones drive structural and functional metabolic reprogramming in fibrotic kidneys by upregulating peroxisome proliferator-activated receptor-γ coactivator-1α (PGC1α), thereby actively restoring impaired renal gluconeogenesis pathways.

To mitigate acute structural damage and cellular stress responses, tanshinones modulate multiple protective protein kinase loops and intracellular receptor complexes. They inhibit the activity of GSK3β and its downstream MAPK signaling networks, while simultaneously coordinating the PI3K/AKT and tumor protein p53 (TP53) pathways to minimize acute tubular necrosis and apoptotic cell death. In conditions of ischemic or drug-induced perfusion loss, these abietane diterpene monomers function as upstream agonists that activate the pregnane X receptor (PXR), which subsequently represses deleterious NF-κB inflammatory networks to preserve baseline renal microvascular blood flow. Under high-glucose conditions, tanshinones block cellular damage by regulating the thioredoxin-interacting protein (Txnip)-mediated activation of the NOD-like receptor family pyrin domain containing 3 (NLRP3) inflammasome (Wu et al. 2023). This specific post-transcriptional bottleneck prevents the downstream activation of caspase-1 and the maturation of interleukin-1β (IL-1β), effectively preventing oxidative stress-induced cell pyroptosis within hyperglycemic environments (Figure S6).

#### Preclinical in vivo validation

The structural execution of these molecular pathways across different renal pathologies has been systematically consolidated into a high-density preclinical matrix. As detailed in Table 6, these distinct in vivo and in vitro configurations map out the precise phenotypic changes, tissue-protective outcomes, and downstream master signaling cascades such as the Txnip/NLRP3 inflammasome, TGF-β/Smad, and PI3K/AKT axes that are targeted by specific naturally occurring abietane diterpene monomers.

**Table 6:** Preclinical renal models, molecular mechanisms, and protective outcomes of tanshinones

Target nephropathy type	Preclinical in vivo model configuration	Active compound/monomer	Phenotypic and tissue-protective outcomes	Key molecular and cellular mechanisms	References
Chronic Kidney Disease (CKD)	Surgical 5/6 nephrectomy rodent model	Tanshinone IIA	Marked improvement in global renal functional parameters Significant reductions in serum creatinine levels Decreased total urinary protein excretion	Suppressed production of inflammatory cytokines Reduction of EC deposition Direct suppression of downstream profibrotic TGF-β/Smad and nuclear factor kappa B (NF-κβ) signaling cascades	Wang et al. 2015a; Nie and Li 2018
Renal fibrosis	Unilateral ureteral obstruction (UUO) and aristolochic acid–induced models	Tanshinone I	Marked reduction in progressive fibrotic tissue remodeling Decreased interstitial collagen accumulation	Target-specific suppression of inhibin beta-A/INHBA, a ligand of the TGF- β superfamily	Wu et al. 2022
Renal fibrosis and metabolism	Obstructed/chemically injured fibrotic kidney models	Tanshinone I	Direct restoration of impaired renal gluconeogenesis pathways Strategic execution of antifibrotic activity via functional metabolic reprogramming	Transcriptional upregulation of peroxisome proliferator-activated receptor-γ coactivator-αPGC1	Wu et al. 2022; Bai et al. 2024
Acute kidney injury (AKI)	Folic acid–induced acute kidney injury	Tanshinone IIA	Alleviation of severe tubular necrosis and cell apoptosis Reduced interstitial inflammatory infiltration Checked downstream progression from acute injury toward chronic kidney disease	Direct inhibition of glycogen synthase kinase-3/GSK3β and downstream mitogen-activated protein kinase MAPK signaling loops Coordinated modulation of PI3K/AKT and tumor protein p53/TP53 signaling clusters	Jiang et al. 2016; Chen et al. 2025
Acute kidney injury (AKI)	Cisplatin-induced acute kidney injury	Tanshinone IIA	Marked improvement in absolute renal blood perfusion Suppressed inflammatory microenvironment injury within renal parenchymal cells	Upstream transcriptional activation of the PXR Subsequent downstream inhibition of deleterious NF-κβ pro-inflammatory signaling pathways	Dou et al. 2022
Diabetic nephropathy (DN)	Streptozotocin (STZ)-induced diabetic nephropathy	Tanshinone IIA	Broad anti-inflammatory and antifibrotic protection Decreased interstitial collagen IV deposition Minimized macrophage tissue infiltration	Direct reduction in the physical abundance of advanced glycation end-products and angiotensin II Suppression of metabolic-driven TGF-β expression cascades	Xu et al.^2020^
Diabetic kidney disease	Genetic hyperglycemic db/db mouse models	Tanshinone IIA	Delayed long-term progression of metabolic renal disease Prevention of oxidative stress–induced cell death	Potent inhibition of Txnip-mediated activation of the NLRP3 inflammasome Downregulation of caspase-1 activation and IL-1β maturation to block inflammasome-driven cell pyroptosis	Wu et al. 2023

#### Translational roadblocks and clinical horizons

Moving beyond animal models, the renoprotective potential of tanshinones has advanced into human clinical valuation as an adjunctive strategy. In randomized controlled trials for hypertensive nephropathy, combining systemic tanshinone with the angiotensin II receptor blocker valsartan significantly improved standard renal markers decreasing serum creatinine, blood urea nitrogen, and microalbumin levels compared to valsartan monotherapy (Chen et al. 2021b). These improvements were accompanied by significant decreases in circulating inflammatory cytokines, oxidative stress parameters, and indicators of endothelial dysfunction, with no serious adverse effects reported, validating a favorable safety profile (Chen et al. 2021b).

However, a major translational bottleneck limits the widespread optimization of these monomers in standard nephrology care. The baseline lipophilic abietane structure of native tanshinones results in exceptionally poor aqueous solubility, restricted intestinal absorption, and rapid phase II first-pass hepatic clearance, which frequently prevents them from attaining therapeutic levels inside targeted renal compartments like the glomerulus and proximal tubules.

To expand these horizons, advanced formulation science is prioritizing target-functionalized nanotechnological delivery systems. For instance, tanshinone IIA–loaded lipid nanocapsules demonstrated vastly superior histological and biochemical protection compared to free monomers in folic acid–induced chronic tubular injury due to enhanced bioavailability, extended plasma half-life, and optimized target accumulation (Neanaa et al. 2025). Moving forward, the clinical success of tanshinones will depend upon these kidney-targeted nanomedicines to protect the core diterpenoid architecture from metabolic clearance and deliver the compounds directly to injured renal epithelial and endothelial cell barriers.

### Effect of tanshinones on liver diseases

#### Molecular and cellular signaling networks

At the molecular level, the multi-target hepatoprotective action of tanshinone monomers is driven by a highly integrated regulatory network that bridges xenobiotic detoxification, antioxidant defense, lipid homeostasis, and anti-fibrotic signaling cascades. A central axis for suppressing acute oxidative and toxicant-induced liver damage is the structural activation of the Nrf2 pathway. Upon activation, Nrf2 drives the transcription of vital downstream cytoprotective and Phase II genes including GCLC, NQO1, and HO-1 which restores endogenous glutathione (GSH) pools and limits lipid peroxidation (Park et al. 2009). To regulate metabolic and fatty infiltration pathologies, tanshinones modulate hepatic lipid metabolism pathways by regulating key nuclear receptors and metabolic enzymes, specifically peroxisome proliferator-activated receptor alpha (PPARα), the wider PPAR signaling pathway, and cytochrome P450 1A2 (CYP1A2). This action normalizes phospholipid metabolism and minimizes ER stress within hepatocytes (Hong et al. 2017; Liu et al. 2022).

To prevent chronic architectural destruction, tanshinones function as potent anti-fibrotic bottlenecks by regulating hepatic stellate cell (HSC) dynamics. They inhibit the TGF-β-stimulated activation of HSCs and suppress extracellular matrix deposition by modulating PI3K/Akt signaling while downregulating Yes-associated protein (YAP) signaling loops. In cholestatic and clearance-failed conditions, these abietane diterpene monomers serve as upstream ligands that activate the PXR. This transcriptional activation upregulates phase I clearing enzymes, including CYP3A isoforms, alongside apical efflux transporters like multidrug resistance protein 1 (MDR1/PRP1), which accelerates toxic bile acid clearance. Furthermore, during systemic inflammatory collapse, tanshinones sustain cellular structural integrity and repress pro-inflammatory cytokine storms by engaging the SIRT1/Sestrin2/HO-1 signaling axis (Figure S7).

#### Preclinical in vivo validation

The structural execution of these molecular pathways across different hepatic pathologies has been systematically consolidated into a high-density preclinical matrix. As detailed in Table 7, these distinct in vivo and in vitro configurations map out the precise phenotypic changes, tissue-protective outcomes, and downstream master signaling cascades targeted by specific naturally occurring abietane diterpene monomers.

**Table 7:** Preclinical hepatic models, molecular mechanisms, and protective outcomes of tanshinones

Target hepatic pathology	Preclinical in vivo/in vitro model configuration	Active compound/derivative	Phenotypic and tissue-protective outcomes	Key molecular and cellular mechanisms	References
Toxicant-induced hepatotoxicity	Carbon tetrachloride (CCl-4) induced acute and subacute models	Tanshinone I, tanshinone IIA, and cryptotanshinone	Significant reduction in serum transaminases Prevented hepatocyte lactate dehydrogenase (LDH) leakage Attenuated systemic lipid peroxidation patterns	Robust restoration of depleted cellular glutathione (GSH) pools Enhanced endogenous antioxidant enzyme activity and suppressed free radical generation	Park et al. 2009
Drug-induced liver injury	Acetaminophen (APAP)-induced hepatotoxicity configurations	Tanshinone IIA	Marked reduction in circulating ALT and AST levels Alleviation of drug-induced oxidative liver injury	Upstream triggering of Nrf2 nuclear translocation Upregulation of downstream GCLC, NQO1, and HO-1 transcriptional activity to neutralize reactive intermediates	Wang et al. 2016
Alcoholic and fatty liver disease	Preclinical ALD and non-alcoholic fatty liver disease (NAFLD) models	Tanshinone monomers	Alleviation of severe hepatic steatosis and inflammatory infiltration Suppression of progressive tissue fibrogenesis	Substantial reductions in tissue malondialdehyde levels Coordinated regulation of PPARα, CYP1A2, and matrix metalloproteinase-2 (MMP2) networks	Hong et al. 2017; Liu et al. 2022
Nonalcoholic steatohepatitis (NASH)	Methionine- and choline-deficient diet model	Tanshinone IIA	Reduced hepatic lipid droplet accumulation Checked inflammatory recruitment and endoplasmic reticulum (ER) stress	Normalization of cellular phospholipid metabolic signatures Targeted regulation of the master PPAR signaling pathway	Xu et al. 2022
Liver fibrosis	Permanent bile duct ligation (BDL) and chronic CCl4 exposure models	Tanshinone IIA	Restriction of parenchymal collagen deposition and scarring Reduction of elevated serum ALT and AST levels Suppression of active hepatic stellate cell (HSC) expansion	Post-transcriptional modulation of PI3K/Akt and Nrf2/HO-1 signaling loops Target-specific inhibition of Yes-associated protein (YAP) signaling to block collagen synthesis	Li et al. 2024; Wang et al. 2025a
Cholestatic liver injury	Lithocholic acid–induced cholestatic models	Tanshinone IIA	Amelioration of severe cholestatic tissue injury Minimization of hepatotoxic bile acid buildup	Upstream transcriptional activation of the pregnane X receptor (PXR) Downstream upregulation of phase I CYP3A isoforms and apical multidrug resistance protein 1 efflux pumps	Ma et al. 2015b
Sepsis-induced liver injury	Cecal ligation and puncture (CLP)–induced polymicrobial sepsis	Tanshinone IIA	Structural preservation of functional hepatic microarchitecture Decreased systemic pro-inflammatory cytokine release	Direct activation of the protective cytoprotective silent information regulator 1 (SIRT1)/Sestrin2/HO-1 signaling axis	Tian et al. 2025

#### Translational roadblocks and clinical horizons

Moving beyond preclinical settings, the hepatoprotective value of tanshinones has successfully transitioned into human clinical evaluation as a specialized adjunctive treatment. In clinical trials evaluating patients with liver cirrhosis complicated by severe acute pancreatitis, combination therapy pairing standard clinical octreotide with STS injection yielded noticeable therapeutic outcomes (Hao et al. 2024). This adjunctive regimen significantly reduced circulating inflammatory biomarkers, optimized systemic endothelial and liver function parameters, shortened total hospitalization duration, and enhanced overall clinical recovery curves without introducing any major safety or off-target toxicity concerns (Hao et al. 2024).

However, a major translational bottleneck continues to limit the widespread optimization of these monomers in standard gastroenterology and hepatology care. The baseline lipophilic abietane structure of native tanshinones results in exceptionally poor aqueous solubility, restricted intestinal absorption, and rapid phase II first-pass hepatic glucuronidation and clearance, which limits their systemic bioavailability when administered in unformulated designs.

To expand these horizons, current formulation science is prioritizing the design of advanced nanotechnological delivery systems. Encapsulating these monomers into liver-targeted nanocarriers such as solid lipid nanoparticles, nanoemulsions, or liposomes surface-functionalized with galactose or asialoglycoprotein receptor (ASGPR) ligands can shield the active diterpenoid structure from premature metabolic breakdown. These advanced platforms allow for the direct delivery of active compounds into parenchymal hepatocytes and activated hepatic stellate cells, optimizing therapeutic outcomes while maximizing local antioxidant and anti-fibrotic actions at reduced doses.

### Effect of tanshinones on bone disorders

#### Molecular and cellular signaling networks

At the molecular level, the therapeutic footprint of tanshinones across skeletal pathologies is defined by a dual regulatory network that decouples bone remodeling by simultaneously suppressing osteoclast-mediated bone resorption and accelerating osteoblast-driven bone formation. The primary antiresorptive checkpoint involves blocking the receptor activator of nuclear factor-κB ligand (RANKL)-driven osteoclastogenesis axis (Cheng et al. 2018; Park and Lee 2025). Tanshinones act as post-transcriptional bottlenecks that interrupt multiple downstream kinase signaling cascades, including the NF-κB, ERK, Akt, JNK, and p38/MAPK.

pathways. Suppressing these master inflammatory loops downregulates crucial osteoclastogenic drivers, downstreams COX-2 expression, and checks local prostaglandin E₂ (PGE) synthesis to halt osteoclast differentiation and limit inflammatory bone destruction. A unique, highly strategic feature of this network is the selective ectosteric inhibition of cathepsin K. Unlike traditional active-site inhibitors, certain tanshinone derivatives alter cathepsin K collagenase activity ectosterically, preserving its essential non-collagenolytic survival functions and providing significant clinical safety benefits (Xu et al. 2021; Sun et al. 2023; Pavanelli et al. 2025; Su and Chen 2025).

Concurrently, these abietane diterpene monomers drive bone anabolism and tissue regeneration by activating osteoblast differentiation networks and maintaining local cellular redox balances. Tanshinones trigger the bone morphogenetic protein (BMP) and canonical Wnt/β-catenin signaling cascades within bone marrow mesenchymal stem cells (MSCs), accelerating their commitment to the osteogenic lineage. They upregulate vital survival factors via Akt1 kinase activation, enhance native cell proliferation, and reverse steroid-induced osteogenesis deficits by scavenging intracellular ROS and blocking osteoblast and osteocyte apoptosis. Under metabolic or diabetic stress, tanshinones counter cellular decay by checking local renin–angiotensin systems and inhibiting the inositol-requiring enzyme 1 alpha (IRE1α)/Txnip/NLRP3 inflammasome axis, which blocks inflammasome-driven cell pyroptosis. Furthermore, they promote localized tissue repair and reverse cell senescence patterns by upregulating phosphoglycerate dehydrogenase (PHGDH) and stimulating osteonecrotic angiogenesis through the induction of SRY-box transcription factor 11 (SOX11) and related vascular markers (Figure S8).

#### Preclinical in vivo validation

The structural execution of these molecular pathways across different bone and skeletal disorders has been systematically consolidated into a high-density preclinical matrix. As detailed in Table 8, these distinct in vivo and in vitro configurations map out the precise phenotypic changes, tissue-protective outcomes, and downstream master signaling cascades such as the Wnt/β-catenin, IRE1\αTxnip/NLRP3 inflammasome, and Nrf2 axes that are targeted by specific naturally occurring abietane diterpene monomers.

**Table 8:** Preclinical bone and skeletal models, molecular mechanisms, and therapeutic outcomes of tanshinones

Target skeletal pathology	Preclinical in vivo/in vitro model configuration	Active compound/monomer	Phenotypic and tissue-protective outcomes	Key molecular and cellular mechanisms	References
Postmenopausal osteoporosis	Ovariectomized rodent models	Tanshinone fractions/tanshinone VI	Complete restoration of alveolar bone microarchitecture Attenuation of bone marrow stromal cell senescence Inhibition of osteoclast differentiation and formation	Targeted upregulation of structural PHGDH networks Suppression of RANKL mediated downstream signaling loops	An et al. 2025
Postmenopausal osteoporosis	Ovariectomized mice and human/mouse osteoclast cultures	Sodium tanshinone IIA sulfonate	Profound in vivo antiresorptive protection Prevention of pathological bone matrix degradation	Selective execution of ectosteric modulation over cathepsin K collagenase activity Preservation of essential non-collagenolytic survival functions	Park and Lee 2025
Steroid-induced osteoporosis	Prednisone- and dexamethasone-induced models (in vivo/in vitro MSCs)	Tanshinol/tanshinone IIA	Successful restoration of bone mineral density Improvement of trabecular microarchitecture Reversal of glucocorticoid-induced osteogenic suppression	Scavenging of toxic intracellular ROS pools Direct activation of Akt1 signaling to enhance MSC proliferation and survival Triggering of BMP and canonical Wnt/β-catenin pathways	Cheng et al. 2018; Wang et al. 2019a
Inflammatory bone loss	LPS-induced bone loss models	Tanshinone IIA	Reduced inflammatory bone resorption Inhibited osteoclast differentiation patterns	Suppression of NF-κβ/ERK/Akt, JNK, and p38/MAPK paths Inhibition of COX-2 expression and PGE synthesis	
Diabetic osteoporosis	Experimental diabetic bone wasting configurations	Tanshinone IIA	Marked improvement in trabecular connectivity and overall bone density Suppression of metabolic-driven bone cellular decay	Localized blockade of the tissue renin–angiotensin system Interruption of the pro-inflammatory IRE1α/Txnip/NLRP3 inflammasome bottleneck to block osteoblast pyroptosis	
Steroid-induced osteonecrosis	Steroid-induced osteonecrosis of the femoral head (SIONFH rodent models)	Tanshinone I	Suppressed oxidative bone cell death Blockade of osteocyte apoptosis Accelerated microvascular neovascularization within the necrotic femoral head	Dual activation of the Nrf2 antioxidant response element and PI3K/Akt/mTOR survival pathways Upstream transcriptional upregulation of the angiogenic factor SOX11 and related vascular markers	Xu et al. 2021
Inflammatory periodontitis	Ligature-induced periodontitis in vivo configurations	Tanshinone IIA and sodium tanshinone IIA sulfonate	Significant limitation of localized inflammatory cell infiltration Preservation of alveolar bone density Prevention of progressive periodontal tissue degradation	Direct downstream inhibition of pro-inflammatory cytokines (IL-1β, IL-17) Downregulation of tissue-destructive matrix metalloproteinase-13 (MMP-13) and cathepsin K expression	Sun et al. 2023; Pavanelli et al. 2025; Su and Chen 2025

#### Translational roadblocks and clinical horizons

Despite the clear multi-target efficacy demonstrated across these preclinical skeletal models, transitioning these monomers into standard human orthopedic care requires overcoming several distinct pharmacological and delivery constraints. The primary translational boundary stems from the highly lipophilic abietane core of native tanshinones, which limits their aqueous solubility and intestinal absorption while subjecting them to rapid first-pass phase II hepatic glucuronidation. Because chronic bone disorders like osteoporosis require prolonged, stable drug exposure, traditional unformulated oral designs face immense hurdles in delivering and maintaining therapeutic monomer concentrations within dense, mineralized bone matrices and poorly vascularized skeletal compartments like the necrotic femoral head.

To expand these clinical horizons, current formulation science is prioritizing the design of advanced nanotechnological drug delivery systems. Encapsulating these monomers into bone-homing polymeric nanocarriers, solid lipid nanoparticles, or liposomes modified with bone-targeting moieties such as tetracycline or bisphosphonate ligands represents a major focus area. These advanced platforms shield the active diterpenoid structure from premature metabolic breakdown and exploit the mineral affinity of the surface ligands to deposit the compounds directly within active bone-remodeling units. Additionally, for localized skeletal pathologies like periodontal bone loss or osteonecrotic lesions, developing bio-absorbable nanofibrous membranes, injectable hydrogels, or smart bone-graft scaffolds loaded with nanoformulated tanshinones offers a promising therapeutic strategy. These localized platforms can bypass systemic clearance loops entirely, providing sustained, controlled release of the active monomer directly into the tissue lesion to optimize anti-inflammatory, antiresorptive, and pro-anabolic bone regeneration programs.

### Effects of tanshinones on CNS disorders

#### Molecular and cellular signaling networks

At the molecular level, the profound neuroprotective capacity of tanshinone monomers principally tanshinone IIA, cryptotanshinone, tanshinone I, and dihydrotanshinone I across diverse neurodegenerative and ischemic pathologies relies on their structural ability to cross the blood–brain barrier and modulate complex neuro-inflammatory, oxidative, and proteinopathic cascades. A central checkpoint in their anti-Alzheimer’s action is the transcriptional upregulation of key amyloid-beta (Aβ)-degrading enzymes, specifically insulin-degrading enzyme and neprilysin, which actively clears neurotoxic peptide plaques. Concurrently, tanshinones serve as post-transcriptional bottlenecks that interrupt the NF-κβ signaling loop. This pathway blockade halts the expression of NF-κβ/Bp65, COX-2, inducible iNOS, and MMP-2, thereby decreasing downstream PGE synthesis and suppressing reactive astrocytosis and microgliosis. This anti-inflammatory profile is further controlled through the long non-coding RNA nuclear-enriched abundant transcript 1 (NEAT1)/microRNA-291a-3p/Rab22a axis, which balances internal Bax/Bcl-2 ratios to halt pro-apoptotic neuronal commitment (Yang et al. 2024).

To mitigate ischemic and dopaminergic decay, tanshinones operate as homeostatic rheostats that govern glial polarization, epigenetic coding, and mitochondrial survival. In ischemic conditions, these monomers direct microglial M1/M2 polarization dynamics, suppressing the neurotoxic M1 phenotype while boosting the anti-inflammatory, tissue-remodeling M2 phenotype. This shifting of the neuro-immunological microenvironment induces an epigenetic regulatory cascade by modifying specific histone acetylation markers, including H3K18ac and H4K8ac (Ma et al. 2022). Simultaneously, tanshinones maintain mitochondrial structural integrity, preserve receptors for activated C kinase 1 (RACK1), and modulate autophagic flux to suppress deleterious hippocampi autophagy. Within dopaminergic pathways, these abietane diterpene monomers prevent proteinopathic aggregation by blocking α-synuclein oligomerization and fibrillation, while upregulating tyrosine hydroxylase expression and activating the Nrf2/HO-1 antioxidant signaling framework to inhibit NADPH oxidase and iNOS-mediated oxidative stress (Figure S9)***.***

#### Preclinical in vivo validation

The structural execution of these molecular pathways across different central nervous system pathologies has been systematically consolidated into a high-density preclinical matrix. As detailed in Table 9, these distinct in vivo and in vitro configurations map out the precise phenotypic changes, neuroprotective outcomes, and downstream master signaling cascades such as the NF-κβ, Nrf2/HO-1, and NEAT1/miR-291a-3p/Rab22a axes that are targeted by specific naturally occurring abietane diterpene monomers.

**Table 9:** Preclinical central nervous system models, molecular mechanisms, and protective outcomes of tanshinones

Target neurological disorder	Preclinical in vivo/in vitro model configuration	Active compound/derivative	Phenotypic and neuroprotective outcomes	Key molecular and cellular mechanisms	References
Alzheimer’s disease (AD)	Transgenic and rodent models of AD	Tanshinone IIA	Dramatic reduction in absolute cerebral Aβ peptide load Suppression of spatial learning and memory deficits Reversal of pathological anxiety behaviors	Transcriptional upregulation of Aβ-degrading enzymes (insulin-degrading enzyme and neprilysin)	Fawzi et al. 2020; Liu et al. 2024b
Alzheimer’s disease (AD)	In vitro cell assays and rat models of AD	Tanshinone IIA	Prevention of Aβ-induced neurotoxicity and neuronal loss Alleviation of active microgliosis and astrocytosis in the temporal lobes and hippocampus	Post-transcriptional inactivation of the NF-κβ B signaling loop Downregulation of COX-2, NF-κβ/p65, iNOS, and MMP-2 expression Decreased downstream synthesis of PGE Suppressed production of pro-inflammatory cytokines (TNF-α, IL-6, IL-1β)	Jiang et al. 2014; Geng et al. 2019; Ding et al. 2020
Alzheimer’s disease (AD)	Rat configurations and cell apoptosis models	Tanshinone IIA	Mitigation of oxidative cell death and neuro-inflammation Prevention of pro-apoptotic neuronal commitment Preservation of baseline spatial memory	Regulation of the long non-coding RNA NEAT1/miR-291a-3p/Rab22a network axis to balance internal Bax/Bcl-2 ratios Restored tissue levels of glutathione and decreased accumulation of malondialdehyde and 8-hydroxy-2′-deoxyguanosine Reversal of depressed SOD kinetics Elevation of hippocampal receptors for activated C kinase 1 (RACK1) to block excessive autophagy	Zhu et al. 2017; Ren et al. 2024; Yang et al. 2024
Cerebral ischemia and stroke	Multi-species stroke and middle cerebral artery occlusion models	Tanshinone IIA	Marked reduction in absolute cerebral infarction volume Attenuation of ischemic tissue edema Protection against apoptotic neuronal damage	Direct modulation of microglial activation and polarization profiles via NF-κβ B loops Suppression of neurotoxic M1 microglia and enhancement of tissue-remodeling M2 microglia	Song et al. 2021
Cerebral ischemia and stroke	Ischemia–reperfusion injury models	Tanshinone IIA microemulsion	Robust neuroprotective benefits despite low baseline water solubility and first-pass elimination	Targeted epigenetic regulatory mechanism driven by controlling the expression of histone acetylation markers H3K18ac and H4K8ac	Ma et al. 2022
Cerebral ischemia and stroke	Oxygen–glucose deprivation and reoxygenation (OGD/R) injury	Sodium tanshinone IIA sulfonate (water-soluble derivative)	Mitigation of acute ischemic neuronal damage Preservation of mitochondrial integrity	Acceleration of survival-linked neuro-autophagy cascades Shifts cytokine balance by suppressing pro-inflammatory (IL-1β, IL-2, TNF-α) and elevating anti-inflammatory (IL-10, TGF-β, BDNF) expressions	Ma et al. 2023
Parkinson’s disease (PD)	Preclinical neurotoxic and nigrostriatal models	Tanshinone IIA	Arrested degeneration of dopaminergic neurons within the nigrostriatal nucleus Suppression of localized oxidative stress	Direct inhibition of iNOS and NADPH oxidase production in the substantia nigra cells	Mohammed et al. 2024
Parkinson’s disease (PD)	Substantia nigra proteinopathy models	Tanshinone monomers	Interruption of PD progression parameters Restored tyrosine hydroxylase expression patterns in the compromised substantia nigra	Strategic activation of the upstream Nrf2/HO-1 antioxidant signaling framework Physical suppression of neurotoxic α-synuclein oligomerization and fibrillation cascades	Hassan et al. 2024

#### Translational roadblocks and clinical horizons

Despite these powerful multi-targeted neuroprotective properties, transitioning native tanshinone monomers into standard human neurological care presents distinct translational boundaries. Although these abietane diterpenes successfully cross the blood–brain barrier in animal models, their native crystalline form is severely restricted by low aqueous solubility, poor intestinal absorption, and intensive first-pass phase II hepatic glucuronidation. These pharmacokinetic constraints create a significant metabolic bottleneck, making it highly challenging to maintain steady, therapeutically effective drug concentrations within human cerebrospinal fluid and targeted deep-brain regions over prolonged treatment periods, as required for chronic conditions like AD and PD.

To expand these clinical horizons, current neuro-formulation strategies are shifting toward advanced, brain-targeted nanotechnological drug delivery systems. Encapsulating tanshinones into advanced nano-vehicles such as lipid microemulsions, solid lipid nanoparticles, or polymeric nanocapsules can shield the active diterpenoid structure from peripheral metabolic breakdown and drastically extend its plasma residence time. To maximize therapeutic outcomes, these advanced nanomedicines can be surface-functionalized with specific blood–brain barrier-homing ligands, such as transferrin, apolipoprotein E, or cell-penetrating peptides. These modifications allow the particles to exploit receptor-mediated transcytosis, ensuring the direct accumulation of active monomers within damaged cortical, hippocampal, and nigrostriatal tissues. Moving forward, developing these advanced nanotechnological platforms represents a vital path to validating the real-world clinical efficacy of tanshinones as multi-targeted neuroprotective agents capable of slowing or reversing human neurodegenerative and ischemic disorders.

### Effects of tanshinones in obesity, type 2 diabetes, and metabolic complications

#### Molecular and cellular signaling networks

At the molecular level, the therapeutic potential of tanshinone monomers against obesity, type 2 diabetes mellitus (T2DM), and their systemic complications is coordinated through a complex regulatory network that balances cellular energy sensors, adipogenic transcription factors, and upstream insulin signaling cascades. A primary mechanism driving anti-obesity and thermogenic actions is the structural activation of the AMPK pathway (Ni et al. 2024). Activation of this metabolic sensor upregulates uncoupling protein 1 (UCP1) and activates essential thermogenic gene networks, promoting brown adipose tissue activity and stimulating the browning of white adipocytes. Concurrently, tanshinones restrict excessive adipogenesis and lipid accumulation by antagonizing PPARγ the master controller of adipocyte development while repressing fatty acid synthase and perilipin A to halt lipid production and storage. This anti-adipogenic effect is further supported by the suppression of STAT3/5 phosphorylation, which breaks down the signal transduction required for pre-adipocyte differentiation. At the epigenetic level, tanshinones alter the anti-inflammatory microenvironment of adipose tissue by regulating specific microRNA expressions; they upregulate protective species like miR-146a-5p and miR-125b-5p to repress NF-κB genes such as RELA and NFKBIA or modulate miR-155-5p to target STAT3-related inflammatory paths (Carpi et al. 2023a).

To counter peripheral insulin resistance and pancreatic decay, tanshinones function as molecular adaptors that restore insulin sensitivity and protect vascular architecture. They target the insulin receptor substrate-1 (IRS-1) pathway, increasing IRS-1 structural stability, augmenting its active phosphorylation, and driving downstream PI3K/Akt survival signaling. This metabolic correction is frequently achieved through the direct inhibition of protein tyrosine phosphatase 1B (PTP1B), a negative regulator of insulin receptor signaling, which enhances glucose uptake. Under high-glucose conditions, activation of AMPK by tanshinones serves as a functional brake that suppresses vascular smooth muscle cell migration and proliferation, while protection within pancreatic islet cells is mediated via the interleukin-6/Janus kinase 2/signal transducer and activator of transcription 3 (IL-6/JAK2/STAT3) signaling cascade (Li et al. 2025b). Furthermore, to limit target-organ complications, tanshinones intercept inflammasome-driven damage by modulating the SIRT1/NLRP3 axis, which blocks oxidative stress–induced cell pyroptosis in the kidneys, suppresses angiogenic VEGF and TNF signaling in the retina, and activates the kinin B2 receptor–Akt–GSK-3β axis in the myocardium. In peripheral tissue repair, they drive macrophage polarization away from the pro-inflammatory M1 state and toward the regenerative M2 phenotype, suppressing NLRP3 inflammasome activation to lower toxic IL-1β and IL-18 secretion (Figure S10).

#### Preclinical in vivo validation

The structural execution of these molecular pathways across different metabolic disorders has been systematically consolidated into a high-density preclinical matrix. These distinct in vivo and in vitro configurations map out the precise phenotypic changes, tissue-protective outcomes, and downstream master signaling cascades such as the AMPK, IRS-1/PI3K/Akt, and SIRT1/NLRP3 inflammasome axes that are targeted by specific naturally occurring abietane diterpene monomers to counteract obesity, type 2 diabetes, and their multi-organ complications. The structural execution of these molecular pathways across different metabolic disorders has been systematically consolidated into a high-density preclinical matrix. As detailed in Table 10, these distinct in vivo and in vitro configurations map out the precise phenotypic changes, tissue-protective outcomes, and downstream master signaling cascades targeted by specific naturally occurring abietane diterpene monomers.

**Table 10:** Preclinical metabolic models, molecular mechanisms, and protective outcomes of tanshinones

Target pathology/complication	Preclinical in vivo/in vitro model configuration	Active compound/monomer	Phenotypic and tissue-protective outcomes	Key molecular and cellular mechanisms	References
Diet-induced obesity	High-fat-diet-induced murine obesity models	Cryptotanshinone	Curtails body weight gain Decreases serum lipid fractions Lowers circulating glucose levels and absolute adipose tissue volume	Activation of the AMPK signaling axis in primary adipose mesenchymal stem cells Halts lipid droplet formation and activates brown adipose genes Upregulation of UCP1-mediated thermogenesis	Ni et al. 2024
Diet-induced obesity	High-fat-diet-induced murine obesity configurations	Tanshinone I	Effectively prevents diet-induced obesity gain Lowers food intake volumes Triggers active browning of white adipose tissue	Systematic activation of the AMPK pathway Upregulation of genes linked to brown adipocyte functional activity in WAT and BAT	Jung et al. 2022
Diet-induced obesity	Animal models of obesity	Tanshinone IIA	Lowers absolute body weight and adipose tissue mass Enhances systematic glucose tolerance Decreases the circulating LDL/HDL cholesterol ratio	Direct antagonism of PPARγ to restrict adipocyte development Suppression of fatty acid synthase and perilipin A Active inhibition of STAT-3/5 phosphorylation to disrupt adipogenesis	Park et al. 2017
Type 2 diabetes (T2DM)	Diabetic rodent configurations	Tanshinone I	Alleviates severe peripheral insulin resistance patterns	Enhancement of baseline IRS-1 protein structural stability Downstream upregulation of protective Akt signaling kinetics	Wei et al. 2017
Type 2 diabetes (T2DM)	Diabetic mouse model panels	Neocryptotanshinone	Increases overall insulin signaling dynamics Accelerates skeletal and hepatic glucose clearance	Marked decrease in protein tyrosine phosphatase 1B (PTP1B) expression Augmentation of IRS-1 phosphorylation and subsequent activation of PI3K/AKT cascades	Hao et al. 2022
Type 2 diabetes (T2DM)	Type 2 diabetic mouse configurations	Tanshinone IIA	Implements broad metabolic protection and glucose tolerance Suppresses high-glucose-induced vascular smooth muscle migration/proliferation Alleviates structural pancreatic islet decay	Upregulation of cellular AMPK energy-sensing activity Regulation of pancreatic cellular integrity via the downstream IL-6/JAK2/STAT3 signaling cluster	Wu et al. 2014; Li et al. 2025b
Diabetic nephropathy	Diabetic kidney damage models	Tanshinone IIA	Reductions in baseline pathological albuminuria Suppression of systemic oxidative and inflammatory injury parameters	Modulation of the internal SIRT1/NLRP3 network axis Reduction of high-glucose-induced cell pyroptosis	Chen et al. 2017; Tian et al. 2026
Diabetic retinopathy	Diabetic microvascular retinopathy models	Tanshinone IIA	Significant optimization and preservation of retinal morphology Prevention of vascular microvascular cell death	Coordinated regulation of hypoxia, oxidative stress, and steroid hormone networks Suppression of angiogenic VEGF, apoptotic PI3K-Akt, and pro-inflammatory TNF pathways	Zeng et al. 2022
Diabetic cardiomyopathy	Experimental diabetic heart failure configurations	Tanshinone IIA (Pre-treatment)	Preservation of myocardial mitochondrial ultrastructure Maintenance of left ventricular systolic contractility Suppression of cardiac cell apoptosis	Target-specific activation of the kinin B2 receptor–Akt–GSK-3β dependent protective pathway	Sun et al. 2011b
Impaired wound healing	Chronic diabetic wound dermis models	Tanshinone IIA	Speeds up functional wound closure and re-epithelialization Promotes architectural collagen organization and angiogenesis	Interruption of the macrophage NLRP3 inflammasome bottleneck to lower toxic IL-1β and IL-18 secretion Accelerates macrophage polarization shifting from M1 to M2 state	Pan et al. 2025

#### Translational roadblocks and clinical horizons

Despite the clear multi-system efficacy demonstrated across these preclinical metabolic models, transitioning these monomers into human clinical therapy presents distinct translational and delivery boundaries. The primary translational bottleneck stems from the highly lipophilic abietane core of native tanshinones, which limits their aqueous solubility and intestinal absorption while subjecting them to rapid first-pass phase II hepatic glucuronidation. Because metabolic disorders like T2DM and chronic obesity require prolonged, stable drug exposure, traditional unformulated oral designs face immense hurdles in delivering and maintaining therapeutic monomer concentrations within target metabolic compartments like expanding white adipose tissue beds, skeletal muscle beds, and the renal cortex.

To expand these clinical horizons, advanced formulation science is prioritizing the design of target-functionalized nanotechnological drug delivery systems. Encapsulating these monomers into smart nano-vehicles—such as solid lipid nanoparticles, nanoemulsions, or liposomes modified with specific targeting moieties represents a major focus area. For instance, surface modifications can be designed to exploit overexpressed receptors on inflamed adipocytes or active endothelial cells. These advanced platforms shield the active diterpenoid structure from premature metabolic breakdown and extend its systemic plasma half-life, allowing for the direct accumulation of active compounds within target tissues to optimize local antioxidant, insulin-sensitizing, and anti-inflammatory mechanisms at reduced doses.

### Skin effects of tanshinones

#### Molecular and cellular signaling networks

At the molecular level, the therapeutic footprint of tanshinones across diverse dermatological pathologies is governed by an interconnected regulatory network that coordinates keratinocyte kinetics, cutaneous lipid metabolism, and macrophage phenotypic polarization. A foundational mechanism in controlling hyperproliferative skin conditions involves enforcing cell cycle arrest and driving programmed cell death in abnormal epidermal layers. Tanshinone monomers target master transcription factors and inflammatory axes, suppressing the systemic interleukin-17/interleukin-23 (IL-17/IL-23) loop and blocking the prostaglandin-endoperoxide synthase 2/nuclear factor kappa B/activator protein 1 (PTGS2/NF-κβ/AP-1) signaling pathways (Fu et al. 2024). This multi-target blockade curtails keratinocyte proliferation by inducing direct G1 phase cell cycle arrest and modulating downstream apoptotic execution cascades. Concurrently, specific monomers serve as explicit post-transcriptional inhibitors of STAT3 activity, which functions as a principal driver of pathological epidermal hyperplasia. To correct abnormal epidermal differentiation, tanshinones re-establish structural homeostasis by normalizing keratin expression signatures, specifically elevating differentiated cytokeratin 1 (K1) abundance while suppressing hyperproliferative cytokeratin 6 (K6) and cytokeratin 17 (K17) levels (Wang et al. 2025b). On the metabolic and microenvironmental fronts, tanshinone monomers actively reprogram cutaneous lipid profiles and govern local macrophage-driven inflammatory responses. They modulate skin lipidomics by correcting disrupted glycerophospholipid and sphingolipid metabolic pathways, which suppresses sebaceous gland hypertrophy and cuts off local lipoplastic inflammatory triggers. Within the dermal microenvironment, these abietane diterpenes intercept inflammatory signaling by blocking the evolutionarily conserved Notch signaling pathway. This specific receptor blockade prevents the polarization of resident macrophages into the pro-inflammatory M1 phenotype, thereby suppressing the release of toxic tissue effectors including inducible iNOS, TNFα, IL-6, IL-20, macrophage migration inhibitory factor (MIF), and monocyte chemoattractant protein-1 (MCP-1). Simultaneously, they promote an anti-inflammatory microenvironment by driving alternative M2 macrophage polarization, which elevates local levels of the mannose receptor (CD206) and IL-4 (Liu et al. 2024a). Furthermore, tanshinones function as robust upstream inducers of the Nrf2 antioxidant response element, executing a critical photoprotective program that shields cutaneous cells from solar-simulated ultraviolet (UV) radiation and associated phototoxic DNA damage (Figure S11).

#### Preclinical in vivo validation

The structural execution of the se molecular pathways across different dermatological conditions has been systematically consolidated into a high-density preclinical matrix. As detailed in Table 11, these distinct in vivo and in vitro configurations map out the precise phenotypic changes, tissue-protective outcomes, and downstream master signaling cascades such as the IL-17/IL-23, STAT3, and Nrf2 axes that are targeted by specific naturally occurring abietane diterpene monomers to counteract psoriasis, acne, and ultraviolet-induced photodamage.

**Table 11:** Preclinical dermatological models, molecular mechanisms, and therapeutic outcomes of tanshinones

Target skin pathology	Preclinical in vivo/in vitro model configuration	Active compound/monomer	Phenotypic and tissue-protective outcomes	Key molecular and cellular mechanisms	References
Plaque psoriasis	Experimental in vivo skin models of psoriasis-like hyperplasia	Tanshinone IIA	Marked suppression of aberrant keratinocyte proliferation Significant reduction in total epidermal thickness Alleviation of localized plaque lesions and dermal inflammation	Downregulation of the IL-17/IL-23 immune cascade Interruption of the pro-inflammatory PTGS2/NF-κβ/AP-1 signaling networks Exhibits strong therapeutic synergy when co-delivered with low-dose methotrexate	Li et al. 2012
Plaque psoriasis	In vivo hyperproliferative skin models	Cryptotanshinone	Robust diminution of psoriatic epidermal hyperplasia Suppression of scaling and cellular overgrowth	Target-specific blockade of STAT3 nuclear translocation and downstream transcriptional activity in keratinocytes	Tang et al. 2018
Psoriasis-like dermatitis	Preclinical dermatitis configurations	Tanshinone I	Suppression of localized inflammatory gene expressions Successful normalization of flawed epidermal architecture	Induction of targeted G1-phase cell cycle arrest and modulation of apoptotic pathways Restored structural cytokeratin K1 levels while checking hyperproliferative K6/K17 markers	Wang et al. 2025b
Inflammatory psoriasis	Animal configurations of inflammatory psoriasis	Tanshinol	Marked limitation of structural skin lesions Shifting of the local immune microenvironment toward a regenerative state	Interception of the evolutionarily conserved Notch signaling pathway Arrest of neuro-inflammatory M1 macrophage polarization Decreased synthesis of iNOS, TNFα, and chemokines Enhanced expression of alternative anti-inflammatory M2 markers (CD206 and IL-4)	Liu et al. 2024a
Acne vulgaris	Preclinical acne models with sebaceous gland hyperactivity	Tanshinone monomers	Marked alleviation of epidermal hyperplasia Suppression of local inflammatory cell infiltration Significant reduction of sebaceous gland hypertrophy	Direct modulation and restructuring of cutaneous lipidomic signatures Reorganization of disrupted cell-surface glycerophospholipid and sphingolipid metabolism	Fu et al. 2024
Ultraviolet photodamage	Newly reconstructed human skin models and in vivo UV irradiation lines	Tanshinone I and dihydrotanshinone	Complete shielding of human skin cells against solar-simulated ultraviolet radiation damage Robust prevention of light-induced apoptotic cascades	Upstream transcriptional activation of the master Nrf2 antioxidant response element Direct neutralization of radiation-induced oxidative stress and prevention of cellular lipid peroxidation	Tao et al. 2013a

#### Translational roadblocks and clinical horizons

Moving beyond animal configurations, the therapeutic potential of tanshinones has successfully transitioned into human clinical evaluation as a highly effective adjunctive strategy for inflammatory skin disorders. In human clinical randomized trials evaluating patients suffering from facial seborrheic dermatitis, combination therapy pairing systemic tanshinone administration with the classic corticosteroid prednisone yielded noticeable therapeutic outcomes (Fu et al. 2022). This combined therapeutic approach drastically reduced clinical skin lesions, target erythema, intensive itching, and desquamation, while concurrently achieving significantly lower adverse event profiles and clinical recurrence rates compared to patient cohorts receiving prednisone monotherapy (Fu et al. 2022). However, despite these compelling clinical and preclinical datasets, a major translational bottleneck continues to limit the widespread optimization of these monomers in standard dermatological and cosmetic practice. The baseline lipophilic abietane structure of native tanshinones results in exceptionally poor aqueous solubility, restricted percutaneous absorption, and rapid phase II first-pass hepatic clearability, which frequently prevents the compounds from attaining stable or sufficient therapeutic levels inside targeted dermal and epidermal skin layers when applied via conventional topical creams or oral designs. To expand these clinical horizons, advanced formulation science is prioritizing the development of novel nanotechnological drug delivery systems. Encapsulating these monomers into smart, target-functionalized vehicles such as lipid microemulsions, solid lipid nanoparticles, ethosomes, or polymeric nanocapsules represents a major focus area. These advanced nanomedicines can seamlessly penetrate the dense stratum corneum barrier, protect the active diterpenoid core from localized enzymatic breakdown, and ensure the sustained, controlled release of active monomers directly within the hair follicles, sebaceous glands, and deep dermal layers. Moving forward, the broad integration of tanshinones into standard clinical dermatology will rely heavily upon these advanced nanotechnological platforms to enhance cutaneous bioavailability, minimize systemic steroid dependence, and exploit the full anti-inflammatory, antiproliferative, and photoprotective traits of these compounds directly at the skin barrier layer.

#### Antimicrobial and antiviral effects of tanshinones

The Gram-positive bacteria, especially drug-resistant strains of *Staphylococcus aureus*, are effectively inhibited by tanshinones (He et al. 2016). Cryptotanshinone demonstrated potent antimicrobial action in vitro against clinical isolates of *S. aureus* bacteria that are resistant to both methicillin and vancomycin. By blocking the action of pyruvate kinase, cryptotanshinone may impede the energy metabolism of bacteria. Combining cryptotanshinone with antibiotics (ampicillin, oxacillin, vancomycin) led to synergistic effects, lowering minimum inhibitory concentrations by more than fourfold against most strains (Cha et al. 2014; Zhong et al. 2021). Cryptotanshinone also has a bacteriostatic activity where it targets the cell membrane and decreases the membrane potential. It inhibits the respiratory chain, most likely by competitively reducing quinone binding to type II NADH:quinone dehydrogenase (NDH-2). It also generates lethal superoxide radicals (Chen et al. 2021a).

*S. aureus*, methicillin-resistant *S. aureus*, and extended-spectrum betalactamases were tested to determine the antibacterial mechanism of dihydrotanshinone I. By causing cell membrane rupture and the release of Fenton-mediated hydroxyl radicals, tanshinone derivatives preferentially inhibit the growth of Gram-positive bacteria. The interaction between the bacterial membranes and these hydrophobic molecules changes the permeability of the membranes, allowing intracellular materials to flow out (Zhao et al. 2021). Besides, tanshinones demonstrated effectiveness, selectivity, and a potential mode of action for treating drug-resistant tuberculosis (Polinário et al. 2025).

As a cap-dependent endonuclease inhibitor, tanshinone I was found to have a wide-ranging antiviral impact. It was highly effective against influenza A, bandaviruses, and arenaviruses in vitro. Tanshinone I considerably decreased viral loads in vivo. The process occurs by attaching to the endonuclease domain of the L protein, which prevents cap cleavage (He et al. 2023).

**Fig. 3:**
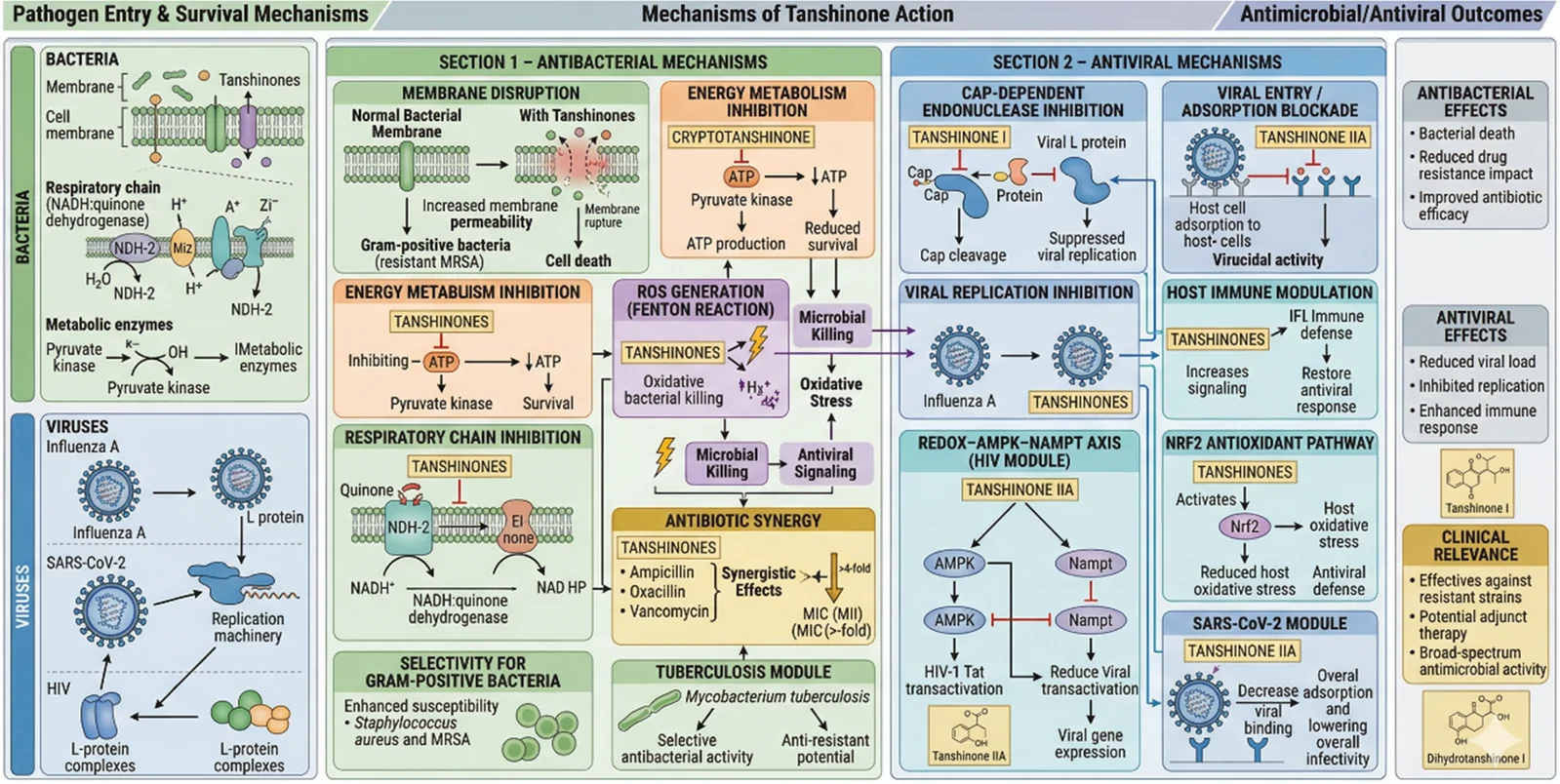
Antimicrobial and antiviral effects of tanshinones. Schematic illustrating broad-spectrum anti-infective mechanisms of tanshinones. The left panel depicts antibacterial activity, including inhibition of Gram-positive bacteria (e.g., MRSA) via disruption of membrane integrity, inhibition of pyruvate kinase and NDH-2, reduced membrane potential, and induction of ROS-mediated damage, with synergistic effects when combined with antibiotics. The central panel highlights antimycobacterial activity through membrane permeability alteration and oxidative stress induction. The right panel shows antiviral effects, including inhibition of viral endonuclease activity (influenza, arenaviruses), suppression of viral entry and replication (SARS-CoV-2), enhancement of IFN-mediated antiviral responses, and modulation of AMPK/Nampt and Nrf2 pathways in HIV and respiratory viral infections. Collectively, tanshinones exert antibacterial, antimycobacterial, and antiviral effects through multi-target mechanisms

Regarding tanshinone IIA, it demonstrated strong antiviral activity against several viruses. For instance, it may impede the influenza A virus’s ability to replicate in vitro and in computational studies. It also can improve antiviral responses and protect against chronic obstructive pulmonary disease caused by cigarette smoke by increasing hemopexin expression in macrophages and lung cells. When administered invitro to cigarette smoke and LPS-exposed lung cells infected with influenza virus, tanshinone enhanced IFN signaling and restored defective antiviral responses (Elebeedy et al. 2022; Yang et al. 2023a). In vitro and computational studies confirmed that tanshinone IIA has anti-SARS-CoV-2 activity. It showed the greatest promise against SARS-CoV-2 in in vitro plaque reduction studies. Although tanshinone IIA mostly inhibited viral adsorption and not replication, it nonetheless demonstrated strong virucidal effects (Elebeedy et al. 2021). Through a redox-regulated AMPK/Nampt pathway, tanshinone II A blocks Tat-induced HIV-1 transactivation. The upregulation of Nrf2 was responsible for reversing the downregulation of glutathione and reactive oxygen species generated. Tanshinone II A blocked the AMPK-Nampt pathway-dependent Tat-induced HIV-1 LTR transactivation (Zhang et al. 2014) (Fig. 3).

## Formulations

### Liposomes

Tanshinone IIA delivery is improved by liposomes. The phospholipid bilayer solubilizes highly lipophilic compounds in the membrane, making insoluble medicines stable water dispersions and increasing apparent solubility and bioavailability. Liposomal encapsulation allows sustained or regulated release, smoother plasma concentration–time profiles, reduced peak-related toxicity, and possible therapeutic improvement. TSIIA showed that liposomes stabilized plasma levels and improved muscle damage and other models. Also, liposomes are biocompatible and have low toxicity. Their composition and surface features, such as PEGylation or ligand conjugation, can be adjusted to extend circulation and tissue-specific targeting, enhancing tanshinone delivery to afflicted areas while limiting off-target exposure (Gatto et al. 2024).

Traditional injectable sustained-release TSIIA liposomes were developed via thin-film dispersion (hydration). The vesicles were small, unilamellar, spherical, and had a uniform size distribution, with an average particle size of 150.67 ± 27.23 nm and a polydispersity index (PDI) of 0.20 ± 0.015, indicating moderate monodispersity. The measured zeta potential was − 8.73 ± 2.28 mV, suggesting a level of constrained electrostatic stabilization. The achieved encapsulation efficiency was 70.32 ± 4.04%, with a drug loading of 15.63%. These liposomes stabilized the highly lipophilic TSIIA in water, improving its solubility compared to the free drug. The in vitro release assays showed an initial burst over 8 h and a total release of 64.46%. After this, a more progressive release phase reached 74.35% at 24 h, consistent with sustained-release behavior. Liposomal TSIIA improved safety and systemic bioavailability by stabilizing plasma levels and reducing peak–trough fluctuations after injection. Compared to free TSIIA, these liposomes reduced inflammation, fibrosis, and oxidative stress and promoted muscle regeneration in a rat model of blunt gastrocnemius muscle damage. This suggests an enhanced pharmacodynamic performance due to improved exposure at the injury site (Wang et al. 2020a).

TSIIA nanocrystal-loaded liposomes (TNC@Lipo) are synthesized using a combined method of antisolvent precipitation and ultrasonic techniques. First, TSIIA nanocrystals are produced. Then, a phospholipid–cholesterol bilayer is applied to enclose the drug core, forming a liposomal shell. The TNC@Lipo hybrid system consists of TSIIA nanocrystals surrounded by a lipid bilayer, achieving a drug loading capacity of 27.86 ± 1.55% and improved colloidal stability over conventional TSIIA liposomes. The formulation is described as nanosized with a uniform distribution, though specific measurements of particle size and zeta-potential are not provided. Transmission electron microscopy shows uniformly spherical or ellipsoidal particles with a distinct core–shell structure. Nanocrystal-liposome systems enhance the solubility of TSIIA by utilizing the high loading capacity of nanocrystals and the stabilizing, water-dispersible properties of liposomes. TNC@Lipo shows a longer drug release in vitro than free TSIIA, attributed to the drug’s diffusion from the nanocrystal core through the lipid shell. TNC@Lipo administered intravenously shows a more effective reversal of liver fibrosis in a mouse model induced by CCl\(4\) than free TSIIA. Evidence shows a reduction in inflammatory mediators like TNF-α and IL-1β, with improved histopathological features, demonstrating superior in vivo efficacy due to enhanced bioavailability and targeted liver delivery (Cai et al. 2025).

Co-delivery liposomes with icariin (ICA) and tanshinone IIA (TSIIA) were modified with the brain-targeting peptide Angiopep-2 (Ang2) for the treatment of Alzheimer’s disease. The multifunctional liposomes were prepared using the thin-film dispersion method. Ang2-modified ICA/TSIIA liposomes were nanosized with a narrow size distribution, mean diameters in the low hundreds of nanometers, and high encapsulation efficiencies for both ICA and TSIIA. Their zeta-potential values indicated stable, mildly negative surface charge, typical for PEGylated or ligand-modified liposomes. Surface conjugation with Ang2 did not significantly alter particle size but provided the liposomes with specific brain-targeting ability. Ang2-decorated liposomes significantly improved transcytosis across in vitro blood–brain barrier models compared to non-modified liposomes, showing better brain penetration. In vitro, the ICA/TSIIA combination demonstrated enhanced cellular uptake in brain microvascular endothelial and glioma cells, resulting in stronger anti-tumor effects when encapsulated in a liposomal carrier. In vivo imaging and pharmacodynamic studies in glioma-bearing mice showed greater brain accumulation, better tumor growth inhibition, and improved survival with Ang2-liposomes compared to control formulations. This indicates that combining liposomal nanocarriers with a BBB-targeting ligand significantly enhances the functional bioavailability of TSIIA in the brain (Wang et al. 2022a) (Figure S12).

### Lipidic nanocarriers

Ashour et al. designed TSIIA-LNCs using a low-energy phase inversion temperature method. They used Labrafac (medium-chain triglycerides), Solutol HS15, Span 80, water, and sodium chloride with controlled heating–cooling cycles between 30 and 55 °C, followed by a cold-water shock to form nanocapsules. Before adding water, TSIIA was dissolved in the molten lipid–surfactant mixture, molecularly dispersing it in the oily core. The TSIIA-LNCs had particle sizes of 60–80 nm, low polydispersity indices (< 0.2), mildly negative zeta potentials (− 10 to − 15 mV), and high encapsulation efficiency (> 90%) and drug loading (mg/mL), indicating efficient entrapment in a small carrier. In vitro, these LNCs generated a stable, transparent dispersion of TSIIA in aqueous conditions, improving its apparent solubility compared to crystalline drug, and displayed a restricted initial burst followed by continuous release over many hours, driven by diffusion and partitioning from the oily core. Oral TSIIA-LNCs showed a 3–fourfold increase in AUC, *C*_max_, half-life, and mean residence time compared to TSIIA solution in rat pharmacokinetic experiments, indicating improved oral bioavailability and extended systemic exposure (Ashour et al. 2020).

Ashour et al. later reported on “modified” TSIIA‑LNCs, where the fundamental LNC platform was adapted for targeted liver delivery through surface modification with phosphatidylserine (PS‑LNCs) or hyaluronic acid (HA‑LNCs). Blank LNCs were prepared using the phase inversion temperature method, incorporating Labrafac, Solutol HS15, Span 80, water, and NaCl. TSIIA-loaded LNCs were produced by dissolving TSIIA (1 mg/mL of the final dispersion) in the oil–surfactant phase prior to processing. PS‑LNCs were synthesized by integrating 0.25–0.5% w/v phosphatidylserine into the initial lipid blend, while HA‑LNCs were created through a post‑insertion/titration method. Initially, cationic LNCs were generated using CTAB to confer a positive charge, followed by a gradual addition to sodium hyaluronate solutions at 37 °C to facilitate electrostatic coating and zeta-potential reversal. Unmodified TSIIA‑LNCs exhibited particle sizes of approximately 70–75 nm with a low PDI of around 0.09. However, elevating the PS concentration to 0.5% w/v resulted in an increase in size and PDI to approximately 86.9 ± 4.9 nm and 0.27 ± 0.03, respectively, while remaining within the nanometric range. The HA coating resulted in a modest increase in size and shifted the zeta potential from positive (CTAB‑LNCs) to negative values, thereby confirming effective surface modification. Encapsulation efficiencies were consistently high at the chosen TSIIA concentration (1 mg/mL), and storage at 4 °C ensured colloidal stability and inhibited drug precipitation for a minimum of 2 weeks. All TSIIA‑LNC formulations significantly enhanced TSIIA dispersibility and apparent solubility relative to the free drug, while maintaining a sustained in vitro release. Variations in release rates were linked to surface composition and diffusional barriers. In vivo, these formulations markedly influenced TSIIA pharmacokinetics: intraperitoneal administration of TSIIA‑LNCs resulted in increased plasma exposure and extended residence time compared to free TSIIA. Additionally, surface‑modified PS‑LNCs and HA‑LNCs further improved liver accumulation and antifibrotic efficacy in a liver fibrosis model, as demonstrated by enhanced biochemical markers and histological findings. Collectively, these studies demonstrate that “plain” TSIIA-LNCs mainly enhance solubility and oral bioavailability, while targeted TSIIA-LNCs maintain these benefits and additionally facilitate ligand-mediated hepatic delivery, resulting in improved therapeutic outcomes (Ashour et al. 2021).

Cryptotanshinone (CTS) loaded solid lipid nanoparticles (SLNs) were formulated to improve the oral bioavailability of cryptotanshinone through ultrasonic and high-pressure homogenization techniques. This method involves melting CTS and solid lipid(s), emulsifying them into an aqueous surfactant phase, and subsequently processing the mixture through high-pressure homogenization and ultrasonication, followed by cooling to produce nanoparticles. The CTS SLNs produced exhibited mean particle sizes of approximately 200 nm, influenced by lipid and sodium deoxycholate content, along with narrow size distributions and negative zeta potentials ranging from −20 to − 30 mV. High entrapment efficiencies were achieved, and the addition of sodium deoxycholate (0.5–1%) enhanced both colloidal properties and loading capacity. The SLNs significantly enhanced the solubility and dispersion of CTS in aqueous media relative to its poorly soluble crystalline form. In vitro studies indicated a sustained release profile compared to drug suspension. In vivo pharmacokinetic studies in rats revealed that CTS SLNs achieved an oral bioavailability of 186–299% relative to CTS suspension, depending on the formulation, and modified CTS metabolism to TSIIA by incorporating the drug into a solid lipid matrix, thereby reducing first-pass metabolism. This study demonstrated that solid lipid nanoparticles (SLNs) significantly improve the oral absorption and metabolic characteristics of a lipophilic tanshinone (Hu et al. 2010).

To enhance circulation time and minimize RES uptake following intravenous administration, “stealth” TSIIA-loaded SLNs (TA SSLNs) were developed using a hot homogenization and ultrasonication process, subsequently coated with Poloxamer 188. The formulation utilized a solid lipid core, such as stearic acid, alongside surfactants, with Poloxamer 188 creating a PEG-like hydrophilic corona on the surface. The TSIIA SLNs produced exhibited mean diameters between 150 and 200 nm, a relatively low PDI, and negative zeta potentials. Additionally, the application of Poloxamer 188 reduced in vitro phagocytosis by macrophages, imparting a “stealth” characteristic. In vitro phagocytosis assays indicated that stealth SLNs were significantly less phagocytosed by macrophages compared to non-stealth SLNs. Additionally, in vivo pharmacokinetic studies in rats revealed that following a single intravenous dose, stealth TSIIA SLNs exhibited a higher AUC and extended circulation time relative to both non-stealth SLNs and TSIIA solution. Consequently, stealth TSIIA SLNs enhanced systemic exposure and residence time, potentially leading to improved therapeutic efficacy for parenteral tanshinone delivery (Zhang et al. 2009).

Zhang et al. developed nanostructured lipid carriers made from high-density lipoprotein (HDL) components to serve as a model system for the lipophilic cardiovascular drug, tanshinone A (TA), employing an ethanol injection/nanoprecipitation method. This design integrates TA into a mixed lipid matrix composed of both solid and liquid lipids, along with HDL-related lipid components and apolipoprotein interactions. The resulting TA-NLCs demonstrated remarkably small mean diameters (approximately 8.0 ± 1.2 nm), a zeta potential of − 29.0 ± 0.0 mV, and a drug loading of 6.17 ± 0.3%, accompanied by high encapsulation efficiencies. These HDL-like NLCs were engineered for extended circulation and to engage with apoA-I and HDL receptors, thus facilitating natural, receptor-mediated transport mechanisms. This paper, while lacking detailed TSIIA data, illustrates that NLC architecture utilizing physiological lipoprotein components can solubilize lipophilic tanshinones and may leverage endogenous lipid transport pathways to improve distribution and bioavailability (Zhang et al. 2010).

To formulate tanshinone ⅡA loaded nanostructured lipid carriers (Tan ⅡA-NLC) and investigate their in vitro transdermal permeation properties, Tan ⅡA-NLC was synthesized using high-pressure homogenization technology and further refined through the Box–Behnken design-response surface method. Its characteristics were analyzed with respect to morphology, particle size, and zeta potential, among other parameters. The assessment of transdermal permeation for Tan ⅡA-NLC was conducted utilizing Franz diffusion cells. The findings indicated that the optimal formulation was characterized by the following parameters: a drug/lipid materials ratio of 88, a GMS/MCT ratio of 2, an emulsifier concentration of 1%, an average particle size of 182 ± 14 nm, a polydispersity index (PDI) of 0.1906 ± 0.0245, and a zeta potential of −27.8 ± 5.4 mV; encapsulation efficiency (EE) was measured at 86.44 ± 9.26% and drug loading (DL) was quantified at 0.98 ± 0.18%, respectively. The in vitro transdermal permeation results indicated that, in comparison to the tanshinone ⅡA solution, Tan ⅡA-NLC exhibited a reduced transdermal permeation amount after 24 h of drug application; however, its retention in the epidermis was 3.18 times greater than that of the solution. The findings suggest that the formulated Tan ⅡA-NLC significantly enhances the retention of tanshinone ⅡA within the epidermis, indicating promising potential for various applications (Zheng et al. 2016).

The co-delivery of DOX and tanshinones is demonstrated through the preparation of lipid nanoparticles loaded with DOX and TAN (N-DOX/TAN), which were synthesized using the emulsification and solvent-diffusion method. A PSMA-targeted ligand was conjugated to N-DOX/TAN to create PSMA-targeted nanoparticles (P-N-DOX/TAN). This system is tested in vitro and in vivo. P-N-DOX/TAN has a zeta potential of 11.2 ± 1.6 mV and dimensions of 139.7 ± 4.1 nm. Compared to N-DOX/TAN, P-N-DOX/TAN released DOX and TAN faster. The synergistic effects of DOX and tanshinone in a single system were confirmed by the fact that N-DOX/TAN inhibited tumor growth more than either N-DOX or N-TAN alone. P-N-DOX/TAN showed the largest uptake in LNCaP cells (58.9 ± 1.9%), highest dispersion in tumor tissue, and highest tumor inhibition effectiveness. Nanomedicine has the potential to deliver therapeutics to prostate cancer cells, enabling synergistic combination therapy (Sun et al. 2021) (Figure S13).

### Polymeric nanoparticles

Tanshinone IIA is found to protect against TGF-β1-induced fibrosis by blocking Smad and non-Smad signaling pathways, preventing myofibroblast activation. We developed inhalable nanoparticles, tanshinone IIA-loaded chitosan-oligosaccharide-coated poly(lactic-co-glycolic acid) (PLGA) nanoparticles (CPN@TAN), to improve pulmonary administration of the drug for pulmonary fibrosis treatment. The 206.5-nm CPN@TAN showed excellent aerosol transport capabilities in vitro, with an MMAD of 3.967 ± 0.025 μm and an FPF of 70.516 ± 0.929%. The nanoparticles were stable throughout atomization and penetrated mucosal barriers better. Confocal spectroscopy confirmed that nanoparticles may transport medicines to NIH3T3, A549, and MH-S cells. In vitro, nanoparticles were biocompatible. In a mouse model of bleomycin-induced pulmonary fibrosis, noninvasive aerosol CPN@TAN inhalation inhibited collagen formation and re-epithelialized the damaged alveolar epithelium without systemic toxicity (Chen et al. 2024).

Tanshinone IIA may treat cerebral ischemia/reperfusion damage. Tanshinone IIA was encapsulated in poly(lactic-co-glycolic acid)-block-poly(ethylene glycol)-carboxylic acid (PLGA-PEG-COOH) nanoparticles to test its efficacy in cerebral ischemia–reperfusion injury (CIRI). Morphological and dynamic light scattering analyses clarified nano-formulations. Dialysis was used to measure medication release. Tanshinone IIA’s cytotoxicity was assessed using the MTT assay on human neuroblastoma cells (SH-SY5Y) and brain endothelial capillary cells (hCMEC/D3). Using oxygen and glucose deprivation/reoxygenation-induced SH-SY5Y/IR cells and a rat model of middle cerebral artery occlusion, the study examined the protective effects of PLGA-PEG-COOH-encapsulated tanshinone IIA on cerebral ischemia–reperfusion injury (CIRI). The study found that PLGA-PEG-COOH-encapsulated tanshinone IIA improved SH-SY5Y/IR cell survival and decreased apoptosis (*P* < 0.01). Tanshinone IIA encapsulation in PLGA-PEG-COOH significantly increased SH-SY5Y/IR cell invasion and reduced inflammation in MCAO rats (*P* < 0.01). Combining PLGA-PEG-COOH-encapsulated tanshinone IIA with angiopep-2 peptide showed a stronger inhibitory impact on CIRI than using it alone (*P* < 0.01). By recognizing lipoprotein-related proteins in brain capillary endothelial cells, the Angiopep-2 peptide helps pass the blood–brain barrier. In conclusion, PLGA-PEG-COOH-encapsulated tanshinone IIA and angiopep-2 peptide may treat cerebral ischemia–reperfusion injury (CIRI) (Zhang et al. 2023b).

Chitosan nanoparticles’ biocompatibility and pH sensitivity make them ideal drug delivery vehicles. We created a solid dispersion of tanshinone IIA and chitosan as the precursor for TA/CS-NPs in this study, a novel and successful technique. This method solved the problem of directly mixing hydrophobic tanshinone IIA and hydrophilic chitosan to make nanoparticles. The response surface methodology determined the optimal TA/CS-NP preparation parameters: 31 mg/mL chitosan, 27:1 CS–TA mass ratio, and 30 min of stirring. In vitro and cellular absorption assays showed pH-sensitive drug release from TA/CS-NPs. Nanoparticles released drugs quickly in pH 5.0 dissolving mediums or inflammatory cells. In the pH 7.4 dissolving medium or normal cell environment, nanoparticle drug release was slow. Cellular tests showed that nanoparticle-delivered tanshinone IIA increased its anti-inflammatory and antioxidant capabilities. TA/CS-NPs are more effective than direct tanshinone IIA in suppressing inflammatory factors such IL-1β, IL-6, TNF-α, and NO. They also regulate oxidative stress products (ROS, MDA) and antioxidant products (SOD, GSH, and CAT) better, protecting mitochondrial damage and suppressing inflammatory signaling pathways (p-p65, NLRP3). This study shows how to encapsulate hydrophobic medicines in hydrophilic chitosan nanoparticles to improve medication efficacy (Luo et al. 2025).

Zhang et al. created lipid–polymeric nanocarriers (LPNs) encapsulating tanshinone IIA. TPGS was covalently bonded to the lipophilic triphenylphosphonium (TPP) cation to form a mitochondrial targeting ligand. After nanoprecipitation, TN-loaded lipid polymer nanoparticles (TN/LPNs) and TPP-TPGS-modified TN/LPNs were assessed in vitro and in vivo against free TN and non-lipid polymeric TN nanoparticles. TN/NPs averaged 89.6 nm and TN/LPNs 121.3 nm, indicating a lipid shell around the polymeric core. The TN/NPs and TN/LPNs zeta potentials were − 33.6 and − 22.3 mV, respectively. TPP-TPGS surface modification reduced negative potential due to positively charged TPP moieties. Free TN and TN/NPs were less compatible and efficacious than TN/LPNs in vitro. TPP-TPGS/TN/LPNs performed better in Sprague–Dawley rats, achieving the highest AUC (129.27 ± 3.37 mg·L⁻^1^·h) and considerable infarct size reduction. The findings suggest that mitochondria-targeted nanocarriers TPP-TPGS/TN/LPNs can efficiently carry TN and other cardiovascular drugs for CVD treatment. Integrating nanoscale dimensions, lipid–polymer hybrid structure, and mitochondrial targeting achieves this goal (Zhang et al. 2018).

A nano-in-micro system that embeds Tan IIA-loaded nanoparticles (Tan IIA-NPs) in EMO-containing microcapsules was designed to overcome to treat renal fibrosis. Sharp flow synthesis method produced homogenous spherical microcapsules with high encapsulation efficiency and drug loading. Cell-penetrating peptides and polyethylene glycol improved the oral bioavailability of Tan IIA by increasing cellular absorption and transmembrane transport in microcapsules with Tan IIA-NPs. This nano-in-micro device also showed sequential drug release. In unilateral ureteral blockage rats, hierarchically organized microcapsules increased EMO and tanshinone ⅡA oral bioavailability and improved therapeutic outcomes. Thus, this research’s nano-in-micro carrier offers a viable oral delivery method for renal fibrosis-targeted combination therapy (Sun et al. 2022) (Figure S14).

### Knowledge gaps and future research priorities

Despite the extensive preclinical evidence mapping the multi-targeted therapeutic potential of tanshinones, several critical knowledge gaps continue to bottleneck their clinical translation. First, a major biochemical contradiction exists within current literature regarding long-term metabolic clearance and cellular safety grids. While native abietane diterpenoid monomers show exceptional efficacy in vitro, their in vivo profiling is severely obscured by rapid phase II hepatic glucuronidation and intestinal P-glycoprotein-mediated efflux, meaning that true steady-state concentrations within deep-tissue compartments (such as the necrotic femoral head or ischemic cortical zones) remain largely unquantified. Furthermore, although semi-synthetic structural modifications like sodium tanshinone IIA sulfonate overcome baseline aqueous solubility limitations, their heightened hydrophilicity introduces a new pharmacokinetic flaw by accelerating rapid renal clearance, which prematurely shortens the therapeutic window. Future research must prioritize the development of advanced biomimetic nanocarriers—such as reconstituted high-density lipoproteins, engineered cell-derived exosomes (ِArif et al. 2026; Ashique et al. 2026) or asialoglycoprotein receptor-targeted lipid nanocapsules—to systematically shield the lipophilic core, bypass systemic first-pass elimination, and achieve site-specific deposition without triggering systemic toxicities. Mechanistically, future investigations must transition away from short-term descriptive phenomenology and instead utilize advanced multi-omics integration and activity-based protein profiling to uncover the precise upstream master genetic rheostats and epigenetic markers that dictate long-term cell survival.

## Conclusion

In conclusion, tanshinones represent a structurally distinctive chemical class of abietane-type diterpenoids exhibiting broad, well-documented therapeutic actions across multiple complex biological systems. Their multi-target bioactivity is tightly controlled through interconnected signaling networks, including the upstream activation of the Nrf2/HO-1 antioxidant axis, the targeted inhibition of hyperproliferative STAT3 and YAP cascades, and the complete suppression of the pro-inflammatory NF-κβ and IRE1α/Txnip/NLRP3 inflammasome bottlenecks. However, severe biopharmaceutical challenges including exceptionally low baseline oral bioavailability, poor aqueous dissolution, and aggressive first-pass metabolic clearance continue to limit their optimization in standard clinical care. Recent breakthroughs in advanced nanotechnology-based drug delivery platforms have begun to successfully address these pharmacokinetic liabilities, offering a promising strategy to optimize tissue targeting and preserve structural stability. Moving forward, the successful evolution of tanshinones from traditional herbal remedies to scientifically validated, targeted single-agent or adjunctive therapies will depend entirely on continuing to bridge the gap between innovative formulation engineering and deep molecular mechanistic research.

## Supplementary Information

Below is the link to the electronic supplementary material.

ESM 1 (23.6 MB DOCX)

ESM 2 (17.9 MB DOCX)

## Data Availability

No datasets were generated or analysed during the current study.

## Abbreviations

AGEs: Advanced glycation end products
Akt: Protein kinase B
AMPK: AMP-activated protein kinase
APAP: Acetaminophen
ATF6: Activating transcription factor 6
Bax: Bcl-2-associated X protein
Bcl-2: B-cell lymphoma 2
Bcl-XL: B-cell lymphoma-extra-large
CAT: Catalase
CGRP: Calcitonin gene-related peptide
CHOP: C/EBP homologous protein
CIPN: Chemotherapy-induced peripheral neuropathy
COX-2: Cyclooxygenase-2
CT: Cryptotanshinone
DHT: Dihydrotanshinone I
DOX: Doxorubicin
EMT: Epithelial–mesenchymal transition
ER: Endoplasmic reticulum
GCLC: Glutamate-cysteine ligase catalytic subunit
GLUT4: Glucose transporter 4
GPx: Glutathione peroxidase
GSH: Glutathione
HO-1: Heme oxygenase-1
HOTAIR: HOX transcript antisense intergenic RNA
ICAM-1: Intercellular adhesion molecule-1
iNOS: Inducible nitric oxide synthase
IRE1α: Inositol-requiring enzyme 1 alpha
IRS-1: Insulin receptor substrate-1
IRS1Ser307: Insulin receptor substrate 1 serine 307 phosphorylation
JNK: C-Jun N-terminal kinase
LC3-II: Microtubule-associated protein 1 light chain 3-II
MAPK: Mitogen-activated protein kinase
MDA: Malondialdehyde
MMP-2: Matrix metalloproteinase-2
MMP-9: Matrix metalloproteinase-9
MRP2/4: Multidrug resistance–associated protein 2/4
mtROS: Mitochondrial reactive oxygen species
MuSCs: Muscle stem cells
NAPQI: *N*-Acetyl-*p*-benzoquinone imine
NDH-2: NADH:quinone dehydrogenase
NEAT1: Nuclear-enriched abundant transcript 1
NGF: Nerve growth factor
NK cells: Natural killer cells
NO: Nitric oxide
NQO1: NAD(P)H quinone oxidoreductase-1
Nrf2: Nuclear factor erythroid 2-related factor 2
p38 MAPK: P38 mitogen-activated protein kinase
PD-1: Programmed cell death protein 1
PD-L1: Programmed death-ligand 1
PERK: Protein kinase RNA-like endoplasmic reticulum kinase
PGE2: Prostaglandin E2
PI3K: Phosphatidylinositol 3-kinase
PPARγ: Peroxisome proliferator-activated receptor γ
RACK1: Receptors for activated C kinase 1
RAGE: Receptor for advanced glycation end products
ROS: Reactive oxygen species
SIRT3: Sirtuin 3
SLC7A11: Solute carrier family 7 member 11
SOD: Superoxide dismutase
TGF-β: Transforming growth factor beta
TRIM28: Tripartite motif-containing 28
UCP1: Uncoupling protein 1
VEGF: Vascular endothelial growth factor
Wnt/β-catenin: Wingless/integrated-beta-catenin signaling pathway
