# Supplementary material for: Unraveling potent therapeutic potentials of tanshinones, lipophilic abietane diterpenes: a holistic literature review
Source: Naunyn Schmiedebergs Arch Pharmacol. 2026 Jul 20;399(13):20215–60. doi: 10.1007/s00210-026-05664-4 (PMC13695630; doi:10.1007/s00210-026-05664-4)
Supplement: Supplementary file 1 [file 210_2026_5664_MOESM1_ESM.docx]

**Figure S1. Therapeutic effects of tanshinones in respiratory system disorders.** Schematic illustrating multi-target actions of tanshinones in asthma, COPD, and acute lung injury. The left panel depicts anti-asthmatic effects, including suppression of Th2 cytokines (IL-4, IL-5, IL-13), inhibition of STAT6 and NF-κB signaling, reduction of eosinophilic infiltration, mucus hypersecretion (MUC5AC), and airway smooth muscle remodeling. The central panel highlights COPD-related mechanisms, showing attenuation of neutrophilic inflammation, inhibition of ERK/p38 MAPK pathways, activation of Keap1/Nrf2 antioxidant signaling, preservation of mitochondrial function, and maintenance of epithelial barrier integrity. The right panel presents protective effects in acute lung injury, including inhibition of TLR4/NF-κB signaling, reduction of pulmonary edema and cytokine production, and activation of Nrf2-mediated antioxidant defenses. Collectively, these mechanisms demonstrate coordinated anti-inflammatory, antioxidant, and tissue-protective effects of tanshinones in respiratory diseases.

**Figure S2. Effects of tanshinones in gynecological disorders.** Schematic illustrating multi-target actions of tanshinones in endometriosis, gynecological cancers, and PCOS. The left panel depicts anti-endometriotic effects, including inhibition of PI3K/Akt/mTOR signaling, suppression of VEGF, HIF-1α, and MMP-9, reduced angiogenesis, inhibition of EMT, and induction of apoptosis (Bax/Bcl-2, caspase-3). The central panel highlights anti-tumor effects in endometrial, ovarian, and breast cancers via activation of p53 pathways, inhibition of SIRT1 and STAT3 signaling, and modulation of estrogen receptor activity. The right panel presents metabolic and hormonal modulation relevant to PCOS, including improvement of insulin resistance, reduction of inflammation and oxidative stress, and regulation of AKT/MAPK and Hippo signaling pathways. Collectively, these mechanisms demonstrate anti-inflammatory, anti-proliferative, anti-angiogenic, and endocrine-modulating effects of tanshinones.

**Figure S3. Anti-cancer and immunomodulatory mechanisms of tanshinones. Schematic illustrating multitarget actions of tanshinones.** The left panel shows direct antitumor effects: inhibition of proliferation *via* PI3K/Akt/mTOR, MAPK, and AMPK pathways; induction of apoptosis through ER stress (PERK/IRE1α/ATF6–CHOP–caspase) and Bax/Bcl-2 modulation; and activation of autophagy *via* AMPK activation and mTOR inhibition (Beclin-1, LC3-II). The central panel highlights anti-metastatic and anti-angiogenic effects, including downregulation of MMP-2/MMP-9, inhibition of EMT, and suppression of VEGF/β-catenin signaling. The right panel depicts immunomodulatory actions, including STAT3 inhibition, M1 macrophage polarization, enhanced dendritic cell maturation, NK cell activation (p38 MAPK), and modulation of PD-L1/PD-1 signaling. Tanshinones also enhance chemosensitivity to agents such as doxorubicin and cisplatin, supporting their potential as adjunct anticancer therapies.

F**igure S4. Protective effects of tanshinones against drug-induced toxicity.** Integrated schematic illustrating multi-organ protective actions of tanshinones. The left panel shows hepatoprotective and nephroprotective effects in acetaminophen toxicity via Nrf2 activation, induction of antioxidant enzymes (HO-1, NQO1), restoration of GSH, and enhanced NAPQI detoxification through MRP2/4. The central panel highlights cardioprotection against doxorubicin through Nrf2 and PI3K/Akt activation, reduced ROS, preserved mitochondrial function, and inhibition of apoptosis (caspase-3, Bcl-XL). The right panel depicts protection against nephrotoxicity, neurotoxicity, ototoxicity, and pulmonary fibrosis via inhibition of TGF-β/Smad signaling, modulation of SIRT3 and SLC7A11, suppression of oxidative stress and inflammation, and regulation of apoptosis (Bax/Bcl-2). Collectively, tanshinones exert antioxidant, anti-inflammatory, anti-apoptotic, and mitochondrial-protective effects.

**Figure S5. Effects of tanshinone IIA in skeletal muscle disorders.** Integrated schematic illustrating multi-target protective actions of tanshinone IIA. The left panel shows attenuation of muscle atrophy via suppression of oxidative stress, mitochondrial ROS, mitophagy, and mitochondrial fission, preserving myofiber integrity. The central panel highlights protection against ischemia–reperfusion injury and fibrosis through inhibition of ICAM-1, TGF-β/Smad3, and PI3K/Akt/COX-2 signaling, with reduced apoptosis and collagen deposition. The right panel depicts enhanced regeneration and metabolic function via activation of MAPK and PI3K/Akt pathways, stimulation of MuSC differentiation (MyoD, MHC), inhibition of adipogenesis (Wnt/β-catenin), and improved glucose uptake (GLUT4, IRS1). Collectively, tanshinone IIA exerts antioxidant, anti-inflammatory, anti-fibrotic, and pro-regenerative effects.

**Figure S6. Effects of tanshinones in kidney disorders.** Integrated schematic illustrating renoprotective actions of tanshinones across CKD, AKI, and diabetic nephropathy. The left panel shows attenuation of renal fibrosis in CKD via inhibition of TGF-β/Smad and NF-κB signaling, reduced ECM deposition (collagen IV, fibronectin), and modulation of INHBA and PGC1α pathways. The central panel highlights protection in AKI through inhibition of GSK3β/MAPK signaling, activation of PI3K/Akt and PXR pathways, suppression of NF-κB–mediated inflammation, and reduction of apoptosis and tubular injury. The right panel depicts mitigation of diabetic kidney disease via suppression of oxidative stress, inhibition of Txnip/NLRP3 inflammasome activation, and reduction of fibrosis, inflammation, and metabolic dysfunction. Clinical evidence indicates improved renal function markers and inflammatory profiles. Collectively, tanshinones exert anti-inflammatory, anti-fibrotic, anti-apoptotic, and metabolic regulatory effects in kidney disorders.

**Figure S7. Effects of tanshinones in liver diseases.** Integrated schematic illustrating hepatoprotective actions of tanshinones across toxic, metabolic, fibrotic, and inflammatory liver disorders. The left panel shows protection against toxicant-induced injury (CCl₄, APAP) via Nrf2 activation, enhanced antioxidant defenses (HO-1, NQO1, GCLC), reduced lipid peroxidation, and preservation of hepatocyte integrity. The central panel highlights mitigation of ALD/NAFLD/NASH through regulation of PPAR signaling, suppression of steatosis, inflammation, ER stress, and normalization of lipid metabolism. The right panel depicts antifibrotic and cholestatic protection via inhibition of TGF-β–mediated stellate cell activation, suppression of YAP signaling, activation of PXR detoxification pathways, and modulation of SIRT1/Sestrin2/HO-1 axis in sepsis-induced injury. Clinical evidence supports improved liver function and inflammatory profiles. Collectively, tanshinones exert antioxidant, anti-inflammatory, antifibrotic, and metabolic regulatory effects.

**Figure S8. Effects of tanshinones in bone disorders.** Schematic illustrating dual regulatory actions of tanshinones on bone remodeling. The left panel depicts antiresorptive effects via inhibition of RANKL-mediated osteoclastogenesis, suppression of inflammatory signaling, and selective ectosteric inhibition of cathepsin K. The right panel highlights anabolic effects through enhancement of osteoblast differentiation, restoration of redox balance, and promotion of bone formation, leading to improved bone mineral density and microarchitecture. Collectively, tanshinones exert balanced antiresorptive and osteogenic effects in osteoporosis, osteonecrosis, and inflammatory bone loss.

**Figure S9.** Neuroprotective effects of tanshinones in CNS disorders. Schematic illustrating multi-target actions in Alzheimer’s disease, cerebral ischemia, and Parkinson’s disease. The left panel shows anti-Alzheimer’s effects, including reduced Aβ accumulation (↑ neprilysin, IDE), inhibition of NF-κB signaling, suppression of neuroinflammation, oxidative stress, and apoptosis. The central panel highlights ischemic protection via modulation of microglial polarization (M1→M2), improved mitochondrial function and autophagy, and reduced neuronal injury. The right panel depicts anti-Parkinsonian effects, including inhibition of α-synuclein aggregation, activation of Nrf2/HO-1 signaling, and preservation of dopaminergic neurons. Collectively, tanshinones exert antioxidant, anti-inflammatory, and neuroprotective effects.

**Figure S10. Effects of tanshinones in diabetes and obesity.** Integrated schematic illustrating multi-target metabolic actions of tanshinones. The left panel depicts anti-obesity effects via AMPK activation, induction of brown adipose thermogenesis (↑UCP1), browning of white adipose tissue, reduced lipid accumulation, and suppression of adipogenesis (PPARγ, STAT3/5). The central panel highlights antidiabetic mechanisms, including enhancement of insulin signaling (IRS-1/PI3K/Akt), inhibition of PTP1B, improved glucose uptake, and modulation of inflammatory pathways (NF-κB, miRNAs). The right panel shows protection against diabetic complications, including nephropathy (SIRT1/NLRP3), retinopathy (VEGF, PI3K/Akt), cardiomyopathy (Akt/GSK-3β), and impaired wound healing via macrophage polarization (M1→M2) and reduced inflammasome activation. Collectively, tanshinones exert metabolic, anti-inflammatory, and insulin-sensitizing effects.

**Figure S11. Skin effects of tanshinones.** Schematic illustrating multi-target dermatological actions of tanshinones. The left panel depicts anti-psoriatic effects via inhibition of IL-17/IL-23, NF-κB/AP-1, and STAT3 signaling, suppression of keratinocyte proliferation, normalization of keratin expression (K1↑, K6/K17↓), and modulation of macrophage polarization. The central panel highlights anti-inflammatory and anti-acne effects through regulation of lipid metabolism (glycerophospholipids, sphingolipids), reduced sebaceous hyperplasia, and decreased inflammatory infiltration. The right panel shows enhancement of skin protection and repair, including Nrf2-mediated antioxidant defense against UV damage and improved outcomes in inflammatory dermatoses. Collectively, tanshinones exert anti-inflammatory, antiproliferative, and photoprotective effects in skin disorders.

**Figure S12. Liposomal formulations of tanshinone IIA.** Schematic illustrating advanced liposomal delivery strategies for tanshinone IIA. The left panel depicts conventional liposomes formed via phospholipid bilayers encapsulating lipophilic TSIIA, enabling improved solubility, sustained release, enhanced bioavailability, and reduced toxicity. The central panel shows nanocrystal-loaded liposomes (TNC@Lipo) with a core–shell structure, combining high drug loading and controlled release with improved stability and enhanced therapeutic efficacy. The right panel illustrates multifunctional ligand-modified liposomes (e.g., Angiopep-2), enabling targeted delivery across biological barriers such as the blood–brain barrier, enhanced cellular uptake, and superior site-specific therapeutic outcomes. Collectively, liposomal systems optimize pharmacokinetics, targeting, and therapeutic performance of tanshinones.

**Figure S13. Lipid-based nanocarriers for tanshinone delivery.** Integrated schematic illustrating advanced lipidic nanocarrier strategies for tanshinones. The left panel depicts lipid nanocapsules (LNCs) with oily cores and surfactant shells, enabling high encapsulation efficiency, improved solubility, sustained release, and enhanced oral bioavailability; surface-modified systems (PS, HA) enable liver-targeted delivery. The central panel shows solid lipid nanoparticles (SLNs) and stealth SLNs, highlighting enhanced stability, controlled release, reduced RES uptake via PEG-like coatings, and prolonged systemic circulation. The right panel illustrates nanostructured lipid carriers (NLCs) and hybrid systems, including HDL-mimetic nanoparticles, transdermal NLCs, and ligand-targeted co-delivery systems, enabling improved tissue targeting, epidermal retention, and synergistic therapeutic efficacy. Collectively, lipidic nanocarriers enhance solubility, pharmacokinetics, targeting, and overall therapeutic performance of tanshinones.

**Figure S14. Polymeric nanoparticle-based delivery systems for tanshinones.** Integrated schematic illustrating advanced polymeric nanocarriers for targeted delivery of tanshinones. The left panel depicts PLGA- and chitosan-based nanoparticles enabling improved solubility, controlled and pH-responsive release, enhanced cellular uptake, and effective pulmonary or systemic delivery (e.g., inhalable CPN@TAN for fibrosis). The central panel highlights PEGylated and ligand-modified systems (e.g., Angiopep-2) facilitating prolonged circulation, reduced inflammation, and efficient blood–brain barrier penetration in cerebral ischemia. The right panel shows hybrid and hierarchical systems, including lipid–polymer nanoparticles with mitochondrial targeting (TPP-TPGS) and nano-in-micro carriers, enabling organ-specific delivery, enhanced bioavailability, sequential drug release, and superior therapeutic efficacy. Collectively, polymeric nanocarriers enhance targeting, stability, and pharmacodynamic performance of tanshinones.
