# Supplementary material for: Unraveling potent therapeutic potentials of tanshinones, lipophilic abietane diterpenes: a holistic literature review
Source: Naunyn Schmiedebergs Arch Pharmacol. 2026 Jul 20;399(13):20215–60. doi: 10.1007/s00210-026-05664-4 (PMC13695630; doi:10.1007/s00210-026-05664-4)
Supplement: Supplementary file 2 [file 210_2026_5664_MOESM2_ESM.docx]

**Table S1. Recent Preclinical Studies on Effects of Tanshinones in as an Anti-inflammatory and Antioxidant Agent.**

| **Tanshinone Type** | **Key Findings** | **Reference** |
| --- | --- | --- |
| Sodium Tanshinone IIA + Sulfonate (STS) | Attenuated vascular senescence in diabetic mice through A20-NFκB-NLRP3 inflammasome-catalase pathway; preserved catalase levels and endothelial function | **(Wei et al., 2024)** |
| Tanshinone IIA | Mitigated postoperative cognitive dysfunction in aged rats by activating hippocampal Nrf2/SLC7A11/GPX4 axis, inhibiting neuroinflammation and ferroptosis | **(Yang et al., 2025)** |
| Tanshinone IIA | Improved cognitive function in Alzheimer's disease animal models; reduced Aβ plaques, neuroinflammation (TNF-α, IL-1β, IL-6), and oxidative stress (ROS, MDA); increased SOD, GSH-Px, and Bcl-2/Bax ratio | **(Rong et al., 2025)** |
| Tanshinone IIA | Suppressed tissue inflammation and fibrosis through PI3K/Akt/mTOR/eNOS, TGF-β1/Smad2/3, NF-κB, JNK/SAPK/MAPK, and ERK/Nrf2 pathways | **(Zhong et al., 2021)** |
| Tanshinone IIA | Demonstrated cardioprotective effects via inhibition of TNF-α/IL-1β signaling, NF-κB, STAT3, and MAPK pathways; enhanced antioxidant enzymes (Cu/Zn SOD, Mn-SOD, GSH) | **(Jin and Li, 2016)** |

Jin, H.-J., and C.-G. J. J. o. C. Li. 2016. Molecular mechanisms of cardioprotective actions of tanshinones. 2016: 9150108.

Rong, Y. et al. 2025. Preclinical evidence and potential mechanisms of tanshinone ⅡA on cognitive function in animal models of Alzheimer’s disease: a systematic review and meta-analysis. 16: 1603861.

Wei, W. et al. 2024. Sodium tanshinone IIA sulfonate alleviates vascular senescence in diabetic mice by modulating the A20-NFκB-NLRP3 inflammasome-catalase pathway. 14: 17665.

Yang, Y., B. Wang, Y. Jiang, and W. Fu. 2025. Tanshinone IIA mitigates postoperative cognitive dysfunction in aged rats by inhibiting hippocampal inflammation and ferroptosis: Role of Nrf2/SLC7A11/GPX4 axis activation. NeuroToxicology 107: 62-73.

Zhong, C. et al. 2021. Recent Research Progress (2015–2021) and Perspectives on the Pharmacological Effects and Mechanisms of Tanshinone IIA. Volume 12 - 2021.
